# Thermal behaviour of lipids in short-lived seeds of Australian rainforest species

**DOI:** 10.1093/aob/mcaf181

**Published:** 2025-08-06

**Authors:** Karen D Sommerville, Lisa Hill, Catherine A Offord, Christina Walters

**Affiliations:** Australian PlantBank, Botanic Gardens of Sydney, Mount Annan, NSW 2567Australia; National Laboratory for Genetic Resources Preservation, United States Department of Agriculture, Fort Collins, CO 80521, USA; Australian PlantBank, Botanic Gardens of Sydney, Mount Annan, NSW 2567Australia; National Laboratory for Genetic Resources Preservation, United States Department of Agriculture, Fort Collins, CO 80521, USA

**Keywords:** Differential scanning calorimetry, *ex situ* conservation, rainforest seeds, seed banking, seed longevity, seed oils, seed storage behaviour, short-lived seeds, triacylglycerols, tropical oils

## Abstract

**Background and Aims:**

Recent studies on desiccation-tolerant Australian rainforest seeds demonstrated that some were short-lived in storage. We sought to understand structural changes of storage lipids that might occur during storage at −20 °C that could contribute to a short lifespan.

**Methods:**

We used differential scanning calorimetry (DSC) to examine exothermic and endothermic transitions during freezing and thawing in dry seed samples of 23 species. Seed samples and extracted triacylglycerols (TAGs) were cooled to −150 °C and rewarmed to 50 °C at 10 °C min^−1^; slower and faster rates of cooling/warming were used for a subset of species to examine lipid crystallization and melting kinetics. Thermograms were analysed for temperature and enthalpy of observed peaks, and these were compared with expected values to detect anomalies. Extracted lipids were further analysed using gas chromatography to characterize fatty acid composition. The thermal profiles of six species were used to design experiments comparing the impact of storage at −20 °C with storage at temperatures outside the range of thermal transitions.

**Key Results:**

Thermal activity was detected in 22 species within the narrow temperature range of −30 and −10 °C; activity at broader temperature ranges was also detected depending on species, cooling protocol and fatty acid composition. A profound interaction between DSC parameters and time at low temperature, as well as fatty acid composition, suggested that TAG crystallization rates contribute to low-temperature sensitivity. We confirmed that damage from TAG crystallization could be avoided by storing seeds at temperatures above TAG crystallization and melting events; storage at cryogenic temperatures improved survival over storage at −20 °C but requires further optimization to maintain pre-storage germination potential.

**Conclusions:**

We conclude that the crystallization and melting of TAGs during storage may negatively impact seed longevity. Seed thermal profiles and rate of TAG crystallization may serve as predictive tools for sensitivity to storage at −20 °C.

## INTRODUCTION

Rainforests comprise a high diversity of plant species (Corlett and Primack, 2008; Sommerville *et al*., 2018) that provide a wide range of useful products and essential ecosystem services (Golden *et al*., 2012; Brandt *et al*., 2014; Delgado-Aguilar *et al*., 2017). This diversity has been declining, and is continuing to decline, under pressure from logging and clearing for agriculture and mining (FAO, 2015; Sommerville *et al*., 2018). Additional environmental stresses such as drought, fire, disease and climate change have led some species to the brink of extinction (Costion *et al*., 2015; Fensham *et al*., 2020; Halofsky *et al*., 2020) and are contributing to a growing need to preserve rainforest species *ex situ*. Seed banking (the storage of dry seeds at low temperatures) is one strategy that may be utilized for this purpose.

To be suitable for long-term seed banking under conditions recommended by the United Nations Food and Agriculture Organization, seeds must tolerate drying at 10–25 % relative humidity (RH) (to obtain a moisture content of 3–7 %) followed by storage at 15 ± 3 % RH and −18 ± 3 °C (FAO, 2014). Modelling by Wyse and Dickie (2017) predicted that 81.5 % of seed-producing species in evergreen rainforests may be tolerant of the drying required for seed banking. That outcome was supported by research on Australian rainforest species, which found that 74 % were tolerant, or at least partially tolerant, of drying to equilibration with 15 % RH; however, a quarter of the desiccation-tolerant species in that study were subsequently found to be very short-lived in storage at −20 °C (Sommerville *et al*., 2021). Other Australian rainforest species have been predicted to be similarly short-lived based on real-time or artificial ageing experiments (Merritt *et al*., 2014; Errington *et al*., 2015; Sommerville *et al*., 2023).

The ability to maintain seed viability in *ex situ* banks is of vital importance, especially for collections of wild seeds, which are generally more difficult to regenerate or re-collect than crop seeds. Re-collection often requires travel to remote locations, sometimes to areas accessible only by foot, or to which access may be limited during the rainy season (when the seeds of some rainforest species are dispersed) or following extreme events such as fires and floods (Martyn Yenson *et al*., 2023). Re-collection of wild species is also impacted by year-to-year variation in seed quantity and quality, ongoing loss of populations due to land clearing, and the incursion of novel pests and diseases that affect seed production. For several rainforest taxa in the Myrtaceae, for example, seed production in the wild is now very limited as a direct result of myrtle rust (Fensham *et al*., 2020; Fensham and Radford-Smith, 2021). Poor longevity of wild seeds stored for conservation purposes is therefore very problematic.

Seeds that tend to age faster than models predict for storage at low water contents and temperatures are generally categorized as ‘intermediate’ in storage behaviour (Ellis *et al*., 1990*a*, *b*; Hong and Ellis, 1996). This behaviour, initially observed in coffee (Ellis *et al*., 1990*a*, *b*), papaya (Ellis *et al*., 1991) and citrus (Hong and Ellis, 1995), has been observed in seeds from tropical areas (Sacandé *et al*., 2000; Dussert *et al*., 2001; Aberlenc-Bertossi *et al*., 2003; Crane *et al*., 2003; Hor *et al*., 2005; Hamilton *et al*., 2009; Chau *et al*., 2019) as well as in oil-rich seeds from temperate tree species (Michalak *et al*., 2013; Ballesteros and Pence, 2017; Williams *et al*., 2023). The prevalence of this seed physiology globally is mostly unknown (Wyse and Dickie, 2017) but its importance to *ex situ* conservation is gaining increasing recognition.

The intermediate seed syndrome has been linked to the thermal behaviour of seed storage lipids (Crane *et al*., 2003; Hamilton *et al*., 2009; Walters, 2015; Ballesteros *et al*., 2019), which provide an important source of energy and carbon during germination and seedling growth (Nikiforidis, 2019). Storage lipids accumulate in cells during seed development and are sequestered from the aqueous cytoplasm in spherical bodies (oleosomes) that are surrounded by a single layer of phospholipids embedded with proteins (such as oleosins or caleosins) (Leprince *et al*., 1998; Murphy *et al*., 2001). The proportion of seed dry mass composed of storage lipids varies greatly among species (SER *et al*., 2023). Seeds that tend to accumulate starch as a food reserve (e.g. cereal grains and pulse crops) may have as little as 2 % lipid per g dry mass (Liu, 2011). In other species, storage lipids may comprise a very high percentage of the dry mass (e.g. 70–80 % in some *Macadamia* varieties; Aquino-Baloños *et al*., 2017).

Seed storage lipids consist of triacylglycerols (TAGs), molecules composed of a three-carbon backbone (glycerol) with a fatty acid linked to each carbon atom. Each of the three fatty acids consists of a chain of carbon atoms joined by single carbon–carbon bonds (saturated) or both single and double bonds (unsaturated). Fatty acid chain length (i.e. the total number of carbons) and degree of unsaturation (i.e. the number of double bonds) affect the phase behaviour of TAGs and are correlated with the temperature and enthalpy of crystallization and melting (Small, 1986; Zeberg-Mikkelson and Stenby, 1999). Phase behaviour, in turn, affects the volume of TAG molecules and the large contraction of oleosome volume upon crystallization has been hypothesized to destabilize cell structure (Walters, 2015; Ballesteros *et al*., 2020), a factor that may contribute to intermediate seed storage behaviour.

Potential links between TAG thermal behaviour and intermediate seed storage behaviour prompted us to investigate the phase behaviour of TAGs in rainforest seeds found to be short-lived in storage at −20 °C. We used differential scanning calorimetry (DSC) to probe temperature ranges that may promote structural instability in dry seeds, which lack freezable water, hypothesizing that thermal activity around −20 °C would be detected and could be attributed to TAGs. We quantitatively characterized the crystallization and melting behaviour of TAGs *in vivo* and in extracted lipids to determine whether these were directly related. We analysed the fatty acid composition of extracted TAGs to determine whether freezing-sensitive species had a high proportion of tropical oils (oils high in saturated and monounsaturated fatty acids that are highly viscous and potentially crystalline at room temperature). Finally, we used the thermal profiles of six species to design experiments comparing the impact of storage at −20 °C with storage at temperatures outside the range of thermal transitions identified by DSC, with the expectation that seed longevity may be improved by the latter.

## MATERIALS AND METHODS

### Species selection and seed source

Twenty-three rainforest species showing intermediate storage behaviour (Sommerville *et al*., 2021) or expected to be short-lived in storage based on artificial ageing (*Syzygium anisatum*, unpubl. data, G. Errington, Botanic Gardens of Sydney, Australia), were selected for investigation. Seed samples for each species had been collected between 2012 and 2015, dried at 15 % RH and stored at −20 °C prior to analysis by DSC in 2016. All seeds were collected from populations in subtropical or warm temperate regions on the east coast of Australia but 11 of the species had a distribution extending to tropical regions (Table 1). Seed samples for each species were withdrawn from storage, thawed, packaged in plastic zip-lock bags and transferred by airmail to the National Laboratory for Genetic Resources Preservation (NLGRP) in Fort Collins, CO, USA, where they were held at ambient humidity (30–40 % RH) prior to analysis.

**Table 1.** Seeds of 23 Australian rainforest species tested for viability while fresh, after drying at 15 % RH and after storage at −20 °C. All but two species showed reduced viability following drying or a brief period of storage; *Pollia crispata* and *Syzygium anisatum* were expected to be short-lived based on artificial ageing experiments. ‘Germ %’ is the final germination percentage, adjusted for empty or missing seeds. Nomenclature is as accepted by the Australian Plant Census, https://biodiversity.org.au. Seeds for all species were collected from subtropical or warm temperate habitats; species in bold text have a distribution extending to the tropics.

		Germination (%)				
Species	Family	Fresh	Post-drying (15 % RH)	Post-storage (−20 °C)	Drying time (months)	Storage time (months)
*Acalypha capillipes*	Euphorbiaceae	nd	100	40	6	2
*Acradenia euodiiformis*	Rutaceae	76	58	28	1	22
***Alphitonia oblata***	Rhamnaceae	96	81	64	6	6
***Archirhodomyrtus beckleri***	Myrtaceae	97	98	24	2	6
***Baloghia inophylla***	Euphorbiaceae	nd	64	12	5	2
***Callicarpa pedunculata***	Lamiaceae	66	54	16	1	44
*Capparis anomala*	Capparaceae	94	44	21	3	43
*Ceratopetalum apetalum*	Cunoniaceae	96	42	26	3	5
*Denhamia silvestris*	Celastraceae	94	72	2	2	25
*Ehretia acuminata*	Boraginaceae	50	12	14	3	34
***Elaeodendron australe* var. *australe***	Celastraceae	70	46	0	3	34
***Emmenosperma alphitonioides***	Rhamnaceae	86	46	28	1	1
***Gynochthodes jasminoides***	Rubiaceae	100	90	3	1	14
***Hymenosporum flavum***	Pittosporaceae	33	91	73	2	22
*Lenwebbia prominens* (2014 collection)	Myrtaceae	94	nd	13	2	6
*Lenwebbia prominens* (2015 collection)	Myrtaceae	79	88	2	3	41
*Lomandra spicata*	Lomandraceae	94	76	63	2	18
***Melastoma affine***	Melastomaceae	nd	92	12	3	47
*Pittosporum multiflorum*	Pittosporaceae	66	65	3	1	1
***Pollia crispata***	Commelinaceae	nd	68	60	7	22
***Polyscias murrayi***	Araliaceae	92	58	22	1	15
*Psychotria daphnoides*	Rubiaceae	67	20	25	1	43
*Rhodamnia maideniana*	Myrtaceae	89	86	0	2	15
*Syzygium anisatum*	Myrtaceae	74	74	73	5	28
	Average	**80.7**	**65.3**	**27.0**	**2.8**	**20.0**
	Standard deviation	**18.3**	**23.9**	**23.7**	**1.8**	**15.0**

nd, not determined.

### Differential scanning calorimetry

Seed samples (3–26 mg dry mass) were hermetically sealed into pre-weighed 20-µL aluminium sample pans (PerkinElmer, Singapore). Depending on seed size, samples consisted of 1–20 whole or partial seeds or, for larger seeds, fractions of embryo alone (for seeds with no endosperm) or embryo plus endospermic tissue. Samples of embryo or embryo plus endosperm were standardized by taking a cross-section from the middle of the seed. Heat flow was measured as the samples were cooled from 20 to −150 °C and then immediately warmed from −150 to 50 °C, usually at 10 °C min^−1^, using one of three DSC 7s (PerkinElmer, Shelton, CT, USA; https://perkinelmer.com). Liquid nitrogen was used as the coolant and helium was used as the purge gas. DSCs were calibrated for temperature with methylene chloride (−95 °C) and indium (156.6 °C), and for enthalpy with indium (28.54 J g^−1^). DSC analysis of seeds from *Pittosporum multiflorum* was conducted later at the Australian PlantBank using a DSC 8000 (PerkinElmer, Singapore) and the same parameters. Additional DSC scans using faster (100 °C min^−1^) and slower (1 °C min^−1^) cooling, as well as a 1-h annealing period during cooling, were conducted for some species. Following thermal analysis, the sample pans were punctured, heated for 24 h at 95 °C and re-weighed to measure dry weight and calculate moisture content.

DSC thermograms were visualized and quantified using Pyris™ software (PerkinElmer, Shelton, CT, USA). Each thermogram plotted heat flow in milliwatts (*y*-axis) versus temperature (°C); exothermic (crystallization) and endothermic (melting) transitions were indicated by downward- and upward-pointing peaks, respectively. The temperatures of crystallization (*T*_crys_) and melting (*T*_melt_) were recorded at each peak minimum or maximum, respectively; the enthalpy of crystallization (*ΔH*_crys_) and melting (*ΔH*_melt_) transitions was calculated from the area under the relevant peak and expressed as J g^−1^ dw. At least three DSC scans using different seeds (i.e. three biological replicates in different pans) were generated for each species.

### Lipid extraction and fatty acid composition

Lipids were extracted from seeds using a modified (Folch *et al*., 1957) method to measure TAG content and composition. Approximately 0.4 g seed dried to ambient RH was ground to a fine powder, weighed, transferred to a large test tube and mixed with 4–5 mL chloroform:methanol (2:1v/v) for ∼10 min, vortexing occasionally. The solvent containing extracted lipids was pipetted into a fresh test tube. The residual ground seed sample was washed two more times with the chloroform:methanol solution to ensure a complete extraction and the solvent fractions from the three washes were combined. The solvent + extracted lipids were mixed with 0.9 % NaCl (0.2 mL mL^−1^ solvent). The lower solvent + lipid phase was then washed twice with 1:1 methanol:0.9 % NaCl solution (0.5 mL mL^−1^ solvent) and transferred by pipette to a pre-weighed test tube. The solvent was evaporated under a stream of N_2_ in a fume hood; the test tube and extracted lipids were then weighed to determine the mass of the total lipid extract. A small sample (0.5–2 mg) of lipid extract was hermetically sealed into a 20-µL aluminium sample pan and analysed by DSC using the same warming and cooling programme applied to intact seeds. Lipid extraction was not attempted for seeds of *Pittosporum multiflorum* or *Syzygium anisatum*.

Extracted lipids were derivatized into fatty acid methyl esters (FAMEs) (Metcalfe and Schmitz, 1961) for analysis by gas chromatography (GC). About 0.2 mL of extracted lipid was mixed with 0.5 mL chloroform and 2 mL of 14 % BF_3_ in methanol (Sigma) in a 5-mL conical reaction vial and heated in boiling water for 3 min. After slight cooling for 2 min, 1 mL water and 2 mL petroleum ether were added; the mixture was shaken and allowed to separate, and the top layer containing FAME was saved. Between 0.25 and 0.5 µL of the petroleum ether FAME solution was separated by GC (Perkin Elmer 8500, Shelton, CT, USA) using a Nukol fused silica capillary column (30 m, 0.25 mm i.d.; Supelco, Burlington, MA, USA). Oven temperature was set at 100 °C and ramped at 10 °C min^−1^ to 190 °C. Injector and detector temperatures (for flame ionisation detection or FID) were set at 220 °C. FAMEs were identified by retention time using multiple FAME standards (Supelco). Proportions of each FAME were quantified by peak area using TurboChrom software (Perkin Elmer 8500, Shelton, CT, USA).

Textbook values given for *T*_melt_ and *ΔH*_melt*_ for the different crystal forms (α, β′ and β) of each fatty acid (Small, 1986; Zeberg-Mikkelson and Stenby, 1999; Cedeño *et al*., 2001; Acree and Chickos, 2025) were weighted by the observed proportions in seeds determined from GC analysis to calculate an expected *T*_melt_ and *ΔH*_melt*_ (J g^−1^ lipid) for TAGs from each species. These were then compared with actual *T*_melt_ and *ΔH*_melt*_ determined by DSC analysis.

### Testing alternative storage conditions suggested by DSC parameters

To confirm sensitivity to storage at −20 °C and test whether storage outside the range of thermal transitions identified by DSC might be beneficial, seeds for six of the target species were re-collected from different locations in 2015 (*Archirhodomyrtus beckleri*) or 2020–21 (*Elaeodendron australe* var. *australe*, *Emmenosperma alphitonioides*, *Melastoma affine*, *Pittosporum multiflorum* and *Rhodamnia maideniana*). Fruits were collected from the wild (*A. beckleri*, *M. affine*, *P. multiflorum*) or from plants with a known wild source cultivated at The Australian Botanic Garden, Mount Annan. Seeds were extracted and cleaned by hand prior to drying. *Archirhodomyrtus beckleri* seeds were dried for 2 months at 20 °C over unsaturated solutions of lithium chloride generating RH of ∼50, 30 and 15 % (Hay *et al*., 2008). Seeds for all other species were held in a drying room for up to 2 months at 15 °C and 15 % RH and removed for testing when the RH surrounding the seeds approximated 30 or 15 % (drying time ranged from 1 week to 2 months). RH for all seed samples was monitored using a hygrometer (model HP23-AW-A) and water activity probe (Rotronic Instrument Corp., Hauppauge, NY, USA; https://www.rotronic.com). Seeds with adjusted moisture were vacuum-sealed into laminated aluminium foil packets then placed into storage at −20 °C and temperatures that were (1) warmer than observed crystallization and melting events, avoiding crystallization altogether (i.e. ≥−5 °C); or (2) colder than observed crystallization and melting events to avoid further changes to crystals formed on cooling (i.e. −192 °C; Table 2). For samples stored at cryogenic temperatures, foil packets were put into a plastic cryostorage box then placed directly into the vapour phase of liquid nitrogen in an MVE 800 series −190 °C storage tank (Chart Industries, Lidcombe). Seeds were withdrawn from storage after 1–12 months, thawed at room temperature (or in a 40 °C water bath for *A. beckleri*), rehydrated at ambient humidity (∼50 % RH) for at least 24 h, then germinated under conditions appropriate for each species (Sommerville *et al*., 2021 supplementary information). Each storage treatment was applied to three to five replicates of 10–20 seeds, depending on the number of seeds available. Seeds were considered germinated on emergence of a healthy radicle and shoot. Storage experiments for *A. beckleri* were conducted twice using the same batch of seeds: once in 2015, soon after collection and prior to DSC analysis; and again in 2022, post-DSC analysis, after the seeds had been held at 4 °C for 7 years.

**Table 2.** Germination following drying and storage for six rainforest species identified as short-lived in storage at −20 °C. Equilibrated RH (%) is the relative humidity to which seeds were dried before storage; Germination (% ± standard error) is the germination percentage after drying and before storage, adjusted for empty seeds. Storage temperatures were selected to re-test response to −20 °C and to test alternative temperatures outside thermal transition zones identified using DSC. The *Archirhodomyrtus beckleri* seed collection was tested twice, once in 2015, soon after collection and prior to DSC analysis, and again in 2022, post-DSC analysis, using the same seed batch which had been stored at 4 °C for 7 years. Storage treatments were applied to three to five replicates of 10–20 seeds per species, depending on the number of seeds available.

	Pre-storage status		Storage conditions	
Species	Equilibrated RH (%)	Germination (%)	Temperature (°C)	Duration (months)
*Archirhodomyrtus beckleri* 2015	Fresh	97 ± 1.7	–	–
	50	95 ± 3.6	4, −20, −192	1
	30	98 ± 2.4	4, −20, −192	1
	15	96 ± 2.6	4, −20, −192	1
*Archirhodomyrtus beckleri* 2022	50	95 ± 3.6	4, −5, −20, −192	1
	15	96 ± 2.6	4, −5, −20, −192	1
*Elaeodendron australe* var. *australe*	Fresh	88 ± 6.1	–	–
	31	97 ± 3.0	15, −2, −20	2
	17	91 ± 5.2	15, −2, −20	2
*Emmenosperma alphitonioides*	Fresh	86 ± 5.1	–	–
	30	96 ± 2.6	15, −2, −20	2
	18	82 ± 7.3	15, −2, −20	2
*Melastoma affine*	Fresh	95 ± 5.0	–	–
	15	79 ± 2.6	−20, −192	12
*Pittosporum multiflorum*	Fresh	95 ± 2.6	–	–
	28	93 ± 2.2	15, −5, −20	1
	17	95 ± 2.6	15, −5, −20	1
*Rhodamnia maideniana*	Fresh	nd	–	–
	15	96 ± 2.6	4, −5, −20, −192	12

nd, not determined.

### Statistical analysis

Combined DSC data from all species were used to calculate mean values (± standard deviation) for temperature of crystallization (*T*_crys_), temperature of melting (*T*_melt_), enthalpy of crystallization (*ΔH*_crys_) and enthalpy of melting (*ΔH*_melt_). Simple linear regressions were used to compare these parameters with (1) water and total lipid content, (2) corresponding DSC parameters of extracted lipids, and (3) predicted DSC parameters based on fatty acid composition.

A non-parametric Mann–Whitney *U* test (*α* = 0.05) was used to test the relative proportions of fatty acid groups (saturated + monounsaturated or polyunsaturated only) for (1) species with a tropical distribution compared with those restricted to subtropical or temperate zones, and (2) seeds that were damaged to some degree by drying (desiccation-intermediate) compared with those that were damaged during conventional freezer storage (temperature-intermediate).

Germination data were arcsine-transformed and analysed using the most appropriate of balanced, two-way or one-way ANOVA (*α* = 0.05) in Minitab v16 (https://www.minitab.com). Residual plots were used to assess whether data met the criteria of normal distribution and equal variance. Non-normal data were analysed using a non-parametric one-way ANOVA (Kruskal and Wallis, 1952) with treatment medians separated by a post hoc Dunn's test. Charts of mean germination percentages (± standard error) were prepared in Microsoft Excel.

## RESULTS

### Thermal behaviour of seed tissues

**Fig. 1.**
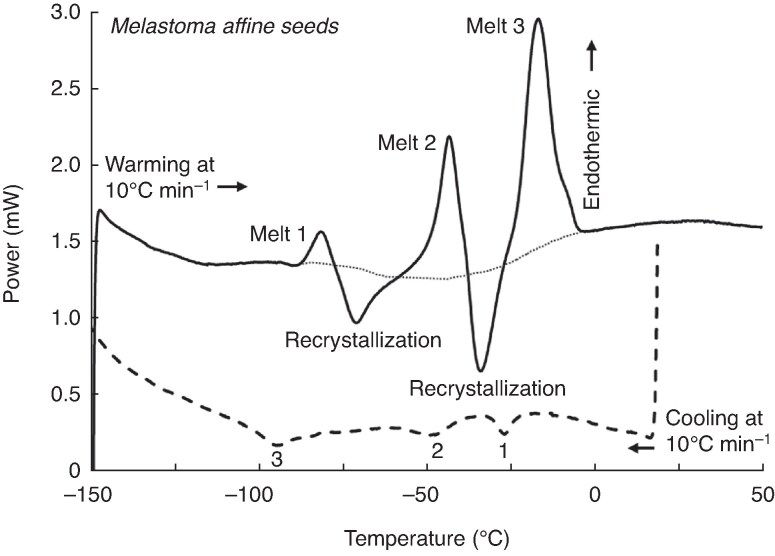
Cooling and warming thermograms generated by DSC for dry *Melastoma affine* seeds showing exothermic (downwards) and endothermic (upwards) events between 0 and −100 °C. Dashed and solid curves represent cooling and warming at 10 °C min^−1^, respectively. The dotted curve is representative of the baseline used to calculate enthalpy of melting (endothermic) events. The thermal activity likely results from crystallization and melting of triacylglycerols; peaks labelled Melt 1, 2 and 3 likely represent various polymorphic crystalline forms.

Thermograms of *Melastoma affine* seeds presented in Fig. 1 illustrate features describing TAG thermal behaviour. The cooling thermogram (dashed curve in Fig. 1) showed small exothermic (crystallization) peaks. For this species, exothermic (downward) peaks upon cooling occurred at −27, −48 and −93 °C and total enthalpy (*ΔH*_crys_) was −1.8 J g^−1^ dw. Cooling thermograms for other species (not shown) were similar, with two or three small exothermic events occurring between ∼−10 and −90 °C; the warmest peak was usually the largest. Among the species tested, crystallization temperature (*T*_crys_) ranged between −10 °C (*Alphitonia oblata*) and −43 °C (*Pollia crispata*), with an average *T*_crys_ of −22.5 ± 7.8 °C (Table 3). Crystallization events upon cooling were largest in terms of enthalpy (*ΔH*_crys_) in seeds of *Capparis anomala*, *Elaeodendron australe* var. *australe* and *Polyscias murrayi* and averaged between −10 and −20 J g^−1^ dw, with an overall average *ΔH*_crys_ among species of −4.6 ± 5.4 J g^−1^ dw (Table 3).

**Table 3.** Thermal parameters measured by DSC for seeds of 23 Australian rainforest species. Seed mass is the average dry weight per individual seed. Seed tissue type is the material used for DSC: whole = whole seeds; Emb + En = embryo + endosperm. *T*_crys_ is the temperature of crystallization for the warmest peak; *T*_melt_ is the temperature of melting for the warmest and largest peaks; *ΔH*_crys_ and *ΔH*_melt_ represent the total enthalpy of crystallization and melting, respectively. Seeds for all species were collected from subtropical or warm temperate habitats; species in bold text have a distribution extending to the tropics.

Taxon	Seed mass (mg ± s.d.)	Seed tissue type	H_2_O content (g g^−1^ dw)	Extracted lipid (g g^−1^ dw)	Calculated lipid (g g^−1^ dw)	T_crys_ (warmest) (°C)	ΔH_crys_ (total) (J g^−1^ dw)	T_melt_ (warmest) (°C)	T_melt_ (largest) (°C)	ΔH_melt_ (total) (J g^−1^ dw)
*Acalypha capillipes*	2.69 ± 0.37	Whole	0.0256	0.32	0.29	−21.4	−1.7	−14.1	−41.4	13.1
*Acradenia euodiiformis*	11.03 ± 3.1	Whole	0.0289	0.32	0.27	−16.9	−9.2	3.9	−17.6	25.3
***Alphitonia oblata***	20.97 ± 3.39	Whole	0.0438	0.06	0.17	−10.1	−0.7	0.2	−20.7	4.9
***Archirhodomyrtus beckleri***	1.25 ± 0.22	Whole	0.0541	0.13	0.11	−28.1	−0.3	−15.4	−15.4	9.2
***Baloghia inophylla***	67.39 ± 23.18	Emb + En	0.0285	0.25	0.27	−24.9	−0.05	4.3	4.3	32.2
***Callicarpa pedunculata***	0.83 ± 0.27	Whole	0.0526	0.08	0.12	−33.4	−0.3	−28.2	−30.6	1.7
*Capparis anomala*	24.69 ± 5.7	Embryo	0.0424	0.24	0.18	−15.4	−10.6	7.1	−10.4	18.5
*Ceratopetalum apetalum*	15.84 ± 5.04	Whole	0.0456	0.19	0.16	−17.8	−6.6	5.5	−11.4	12.3
*Denhamia silvestris*	11.12 ± 2.63	Emb + En	0.0231	0.32	0.3	−22.2	−8.8	−11.6	−22.2	9.0
*Ehretia acuminata*	12.43 ± 2.66	Whole	0.0481	0.07	0.15	−27.5	−0.1	−32	−32	1.1
***Elaeodendron australe* var. *australe***	254.5 ± 34.7	Emb + En	0.0054	0.45	0.41	−17.7	−14.7	−6.2	−18.9	19.1
***Emmenosperma alphitonioides***	38.98 ± 1.72	Emb + En	0.0529	0.06	0.12	−12.6	−3.1	5.4	−20.7	12.2
***Gynochthodes jasminoides***	4.83 ± 0.81	Whole	0.0507	0.1	0.13	−15.6	−1.4	−12.1	−37.2	5.3
***Hymenosporum flavum***	3.62 ± 0.86	Whole	0.0805	0.04	0.01	−19.8	−7.5	−0.2	−0.2	10.9
*Lenwebbia prominens*	9.03 ± 3.68	Whole	0.0543	0.04	0.11	−32.8	−0.7	−14.1	−14.1	7.2
*Lomandra spicata*	13.11 ± 6.28	Whole	0.0535	0.1	0.11	−32.6	−1.9	−14.1	−32.1	5.2
***Melastoma affine***	0.06 ± 0.01	Whole	0.0465	0.19	0.16	−26.6	−1.8	−16.4	−16.4	20.2
*Pittosporum multiflorum*	13.24 ± 0.92	Half seed	0.0653	nd	0.04	−15.8	−5.3	28.7	−1.6	9.8
***Pollia crispata***	0.81 ± 0.07	Whole	0.081	0.01	0.01	−42.7	0	−13	−22.7	0.02
***Polyscias murrayi***	2.62 ± 0.39	Whole	0.0451	0.2	0.17	−19.4	−20.3	24.8	24.8	30.3
*Psychotria daphnoides*	11.48 ± 2.41	Whole	0.059	0.04	0.08	−17.2	−1.7	−16.5	−29.1	9.8
*Rhodamnia maideniana*	15.45 ± 5.68	Whole	0.0464	0.1	0.16	−26.4	−1	−14.8	−24	11
*Syzygium anisatum*	4.75 ± 1.48	Embryo	0.0319	0.28	0.25	−20	−7.2	9.3	−18.6	22.9
Average	**13.01 ± 3.22**		**0.0463**	**0.16**	**0.16**	**−22.5**	**−4.6**	**−5.2**	**−17.7**	**12.7**
Standard deviation	**15.29 ± 4.88**		**0.0174**	**0.12**	**0.1**	**7.8**	**5.4**	**15**	**14.7**	**8.9**

nd, not determined.

Endothermic peaks from the warming thermogram of the same sample of *Melastoma affine* (solid curve in Fig. 1) occurred at temperatures about 10 °C warmer (−80, −42 and −16 °C) than the exotherms observed on cooling. Upon warming, there were two additional exothermic peaks at −70 and −33 °C that have been labelled as recrystallization events. These likely indicate TAG molecules that did not crystallize during cooling. The dotted curve from −95 to 0 °C represents a curvilinear baseline, above and below which enthalpy of melting and crystallization, respectively, were calculated. The total melting enthalpy (*ΔH*_melt_) of the sample in Fig. 1 was 20.2 J g^−1^ dw, a 10-fold increase from *ΔH*_crys_. The difference in *ΔH*_crys_ and *ΔH*_melt_ suggests that crystal growth can occur during warming under the experimental conditions of 10 °C min^−1^ cooling and warming.

**Fig. 2.**
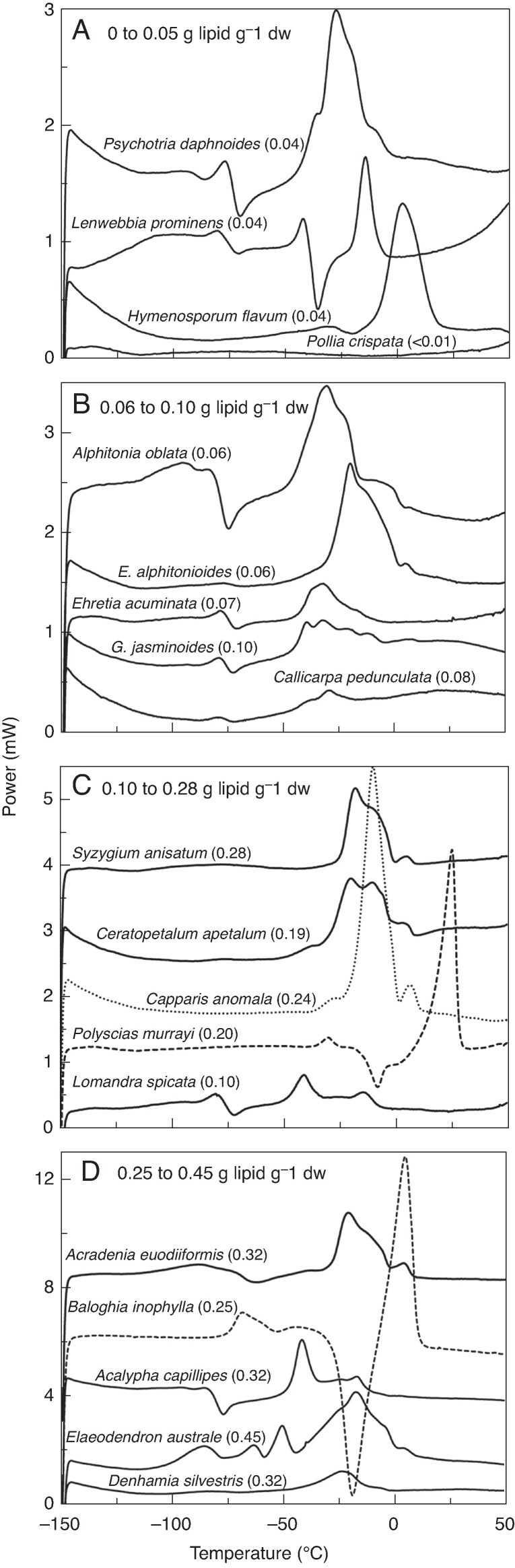
Representative thermograms for seeds of 19 Australian rainforest species grouped by lipid content ranging from 0 to 45 g lipid g^−1^ dw (Table 2). Exothermic (downward pointing) and endothermic (upward pointing) peaks indicate crystallization and melting events, respectively. The *y*-axis scale increases from 3 to 13 mW between panels (A) and (D), confirming lipid content is a factor in the size of transitions. Taxon names are shown immediately above the relevant thermograms; figures in brackets represent percentage seed lipid content (dry mass basis).

**Fig. 3.**
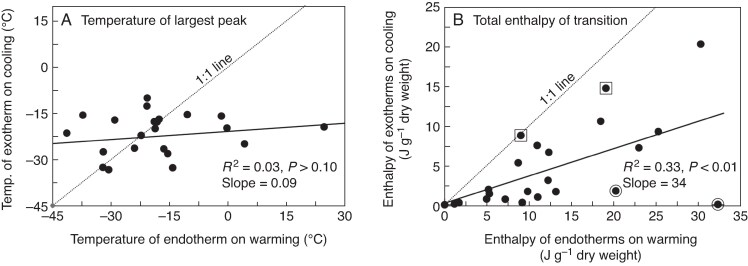
Relationships between temperature (A) and enthalpy (B) of TAG transitions measured in seeds of Australian rainforest species from warming (x-axis) and cooling (*y*-axis) thermograms. Correlation models show poor correspondence between cooling and warming transitions. The encircled outlying points in (B) represent *Baloghia inophylla* and *Melastoma affine*, while the boxed points represent two Celastraceae species, *Elaeodendron australe* var. *australe* and *Maytenus silvestris*, the latter being represented by the point on the 1:1 line.

Complex thermal behaviours between −25 and 0 °C were apparent from warming thermograms of seeds from most of the study species (Fig. 2). Seeds of *Pollia crispata* displayed negligible thermal events between −150 and +50 °C, consistent with its very low lipid content of 0.01 g g^−1^ dw (Fig. 2A). The transitions within *Baloghia inophylla* seeds (Fig. 2D, dashed curve) were unusually large and sharp, despite negligible activity observed on cooling (Table 3; Fig. 3B, encircled point at 32 J g^−1^ on the *x*-axis). The temperature of the largest melting peak (*T*_melt_) varied among species from above 0 °C (*Polyscias murrayi*, 24.8 °C, Fig. 2C; *Baloghia inophylla*, 4.3 °C, Fig. 2D) to below −37 °C (*Gynochthodes jasminoides*, −37.2 °C, Fig. 2B; *Acalypha capillipes*, −41.4 °C, Fig. 2D) with an average *T*_melt_ of −17.7 ± 14.7 °C (Table 3). Enthalpy of melting for the 23 species ranged from near 0 (*Pollia crispata*) to 32 J g^−1^ dw (*Baloghia inophylla*), with an average *ΔH*_melt_ of 12.7 ± 8.9 J g^−1^ dw (Table 3).

**Fig. 4.**
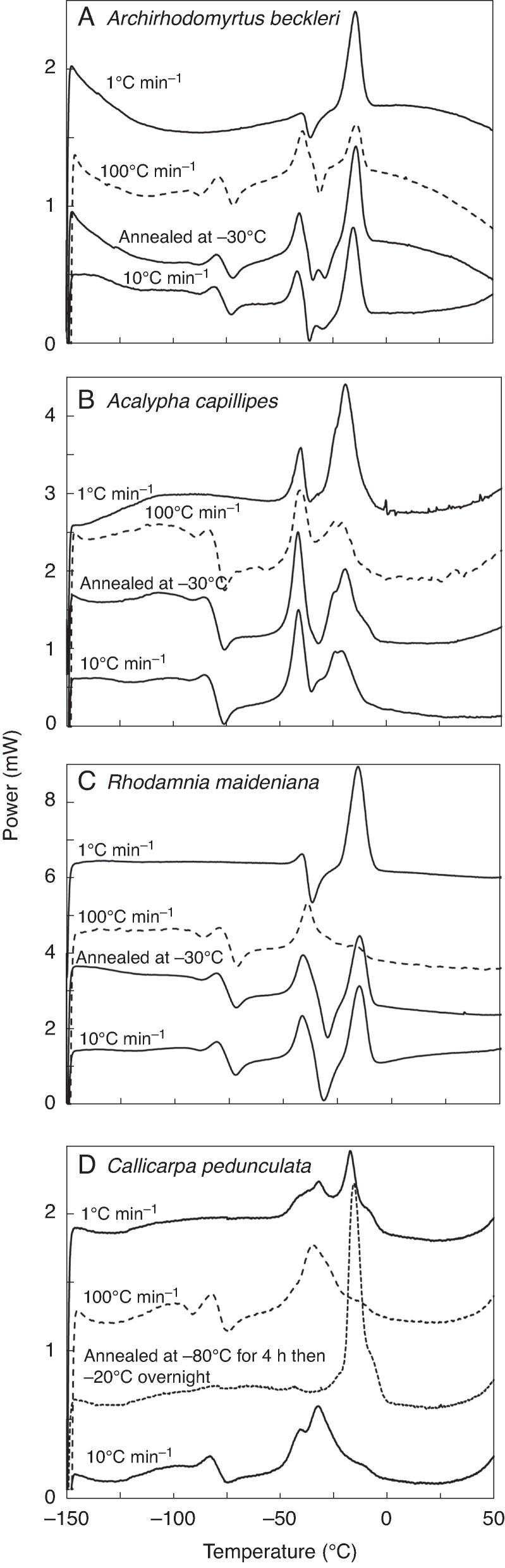
Effect of cooling rate, and a period of annealing during cooling, on warming thermograms of whole seeds of (A) *Archirhodomyrtus beckleri*, (B) *Acalypha capillipes*, (C) *Rhodamnia maideniana* and (D) *Callicarpa pedunculata.* Exothermic (downward pointing) and endothermic (upward pointing) peaks indicate crystallization and melting events, respectively. The period of annealing consisted of 1 h at −30 °C for species in (A–C) and 4 h at −80 °C followed by overnight storage at −20 °C for *C. pedunculata*.

Temperatures for crystallization and melting transitions among seeds from different species were poorly correlated (Fig. 3). While melting temperature ranged broadly (see the largest endothermic peaks presented in Figs 1, 2 and 4), peak temperatures for crystallization during cooling were confined to a narrow temperature range, usually below −15 °C (Fig. 3A). The poor correlation between crystallization and melting temperatures (*r*^2^ = 0.03; Fig. 3A), and quite low intercept (−21 °C) and slope (0.09), suggested that substantial supercooling (i.e. cooling below the melting temperature, which usually reflects equilibrium, *without* crystallization) may have occurred before crystallization. Low levels of crystal formation during cooling were also reflected by low values of *ΔH*_crys_, which were on average 2.8-fold less than values of *ΔH*_melt_ measured during warming (compare averages of −4.6 and +12.7 J g^−1^ dw for cooling and warming, respectively; Table 3). These comparisons suggested that crystallization was incomplete during cooling to −150 °C using a 10 °C min^−1^ protocol.

### Effect of cooling rate and annealing on DSC parameters

Hypothesizing that slow crystallization explained the poor correlation between thermal behaviours during cooling and warming (Fig. 3), we adjusted cooling rate in a subset of seeds to see if this changed thermogram shape or *ΔH*_melt_ size. Faster or slower cooling (at 100 or 1 °C min^−1^) tended to reduce or increase, respectively, the prominence of the largest melting peaks (Fig. 4). Slower cooling also reduced or eliminated recrystallization events (exothermic events upon warming in Figs 1 and 2). An annealing treatment, in which samples were cooled at 10 °C min^−1^ and held at −30 °C for 1 h before cooling further to −150 °C did not affect thermogram shape but seemed to increase *ΔH*_melt_ in some cases (not quantified here). The annealing protocol for *Callicarpa pedunculata* (Fig. 4D) involved a pretreatment at −80 °C to nucleate crystals and then overnight storage at −20 °C. This treatment resulted in a profound increase in both *T*_melt_ and *ΔH*_melt_ compared with the original 10 °C min^−1^ cooling protocol.

### Thermal behaviour of extracted lipids

**Fig. 5.**
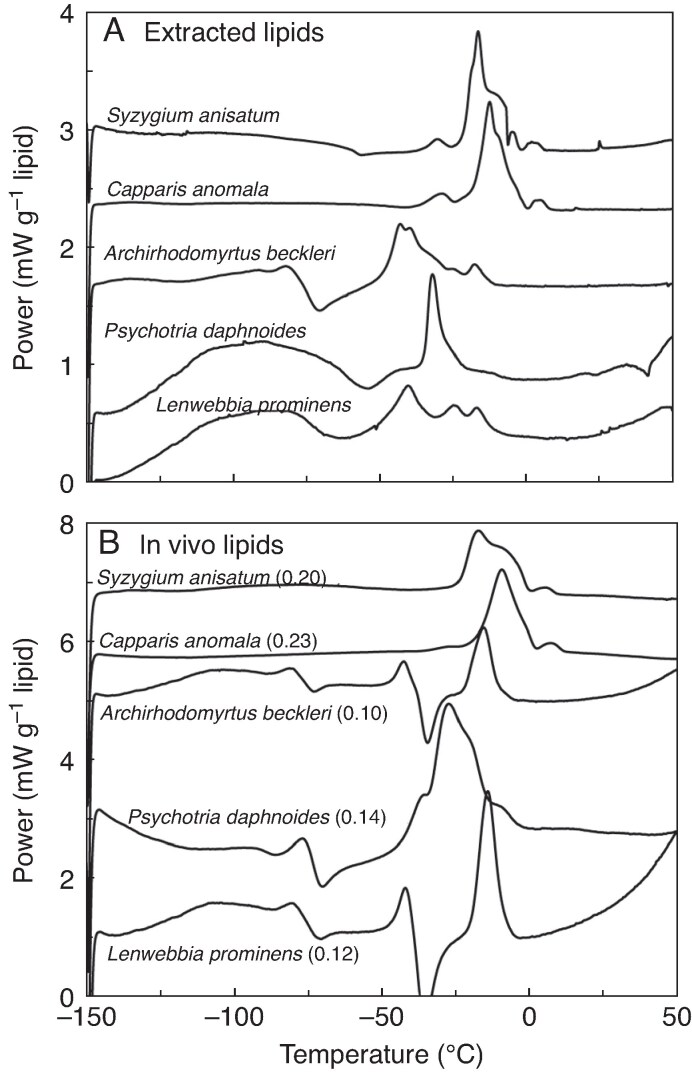
DSC thermograms of lipids extracted from seeds (A) and thermograms of the associated seeds (B). Exothermic (downward pointing) and endothermic (upward pointing) peaks indicate crystallization and melting events, respectively. The power *y*-axis for the thermograms is corrected by the amount of lipid in the sample, determined in (A) directly from the mass of the sample and in (B) by dividing the sample mass by the proportion of lipid comprising the dry mass. Note that the ordinate in (B) is twice as large as in (A).

**Fig. 6.**
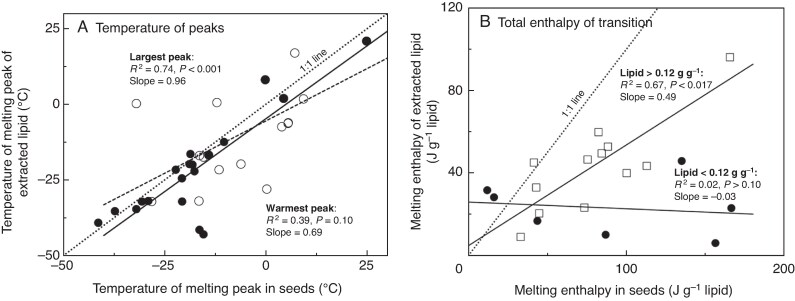
The relationship between melting temperature (A) and enthalpy of melting transitions (B) measured in seed tissues (*x*-axis) and extracted lipids (*y*-axis). A 1:1 line has been added as an aid to the eye. In (A), data are expressed as the temperature of the largest melting peak (solid circles, solid line) or the warmest melting peak (open circles, dashed line) in thermograms generated by DSC; the results of regression models are presented for each. In (B) seeds with lipid content less than and greater than 0.12 g lipid g^−1^ dry weight are designated by solid and open symbols, respectively. Enthalpy of melting (*ΔH*_melt*_) of *in vivo* and extracted lipids was correlated for oily seeds, but *ΔH*_melt*_ of extracted lipids was about half the amount observed *in vivo*. For seeds containing <0.12 g lipid g^−1^ dry weight, *ΔH*_melt*_ of extracted lipid varied little while *ΔH*_melt*_in vivo varied broadly.

Thermograms of lipids extracted from seeds (Fig. 5A) resembled those of whole seeds (Fig. 5B) from the same species, though there were differences in shape and recrystallization events. For extracted lipids, the temperature of the largest melting peak ranged from −43 °C (*Archirhodomyrtus beckleri*) to +21 °C (*Polyscias murrayi*) with an average *T*_melt_ of −21.7 ± 17.0 (Fig. 6A; Supplementary Data Table S1). The temperatures of the most prominent melting peak in extracted TAGs and whole seeds were strongly correlated (Fig. 6A, solid circles; *r*^2^ = 0.74, slope = 0.96; *P* < 0.001). The temperatures of the warmest-melting peaks in extracted lipids and whole seeds were not correlated (Fig. 6A, open circles).

Melting enthalpy of extracted lipids (*ΔH*_melt*_) ranged from 5.9 (*Hymenosporum flavum)* to 96 (*Polyscias murrayi*) J g^−1^ lipid with an average *ΔH*_melt*_ of 35.7 ± 21.5 J g^−1^ lipid. To compare melting enthalpies of TAGs extracted from seeds and TAGs *in vivo*, we divided *ΔH*_melt_ (J g^−1^ dw) by lipid content (g lipid g^−1^ dw; Table 3) to obtain *ΔH*_melt*_ (J g^−1^ lipid) for TAGs *in vivo*. The value for *ΔH*_melt*_ ranged from 3.6 (*Pollia crispata*) to 166.4 (*Psychotria daphnoides*) J g^−1^ lipid with an average *ΔH*_melt*_ of 79.6 ± 46.7 J g^−1^ lipid. Values of *ΔH*_melt*_ for seeds and extracted lipids were correlated for seeds containing >0.12 g lipid g^−1^ dw (Fig. 6B, open squares; *r*^2^ = 0.67, *P* < 0.017, *n* = 12); however, the slope of 0.49 was well below an expected 1:1 relationship, and indicated about twice as much lipid crystallized *in vivo* than in extracted samples using our cooling/warming protocols. This trend was exacerbated in seeds with lower lipid contents (<0.12 g lipid g^−1^ dw) such that there was no significant correlation between *ΔH*_melt*_ for *in vivo* and extracted lipids (Fig. 6B, solid circles; *r*^2^ = 0.02, *P* > 0.10, slope = −0.03, *n* = 7). Species with low lipid contents and unexpectedly high melting enthalpies were *Psychotria daphnoides*, *Hymenosporum flavum* and *Emmenosperma alphitonioides*.

### Lipid content and fatty acid composition

**Fig. 7.**
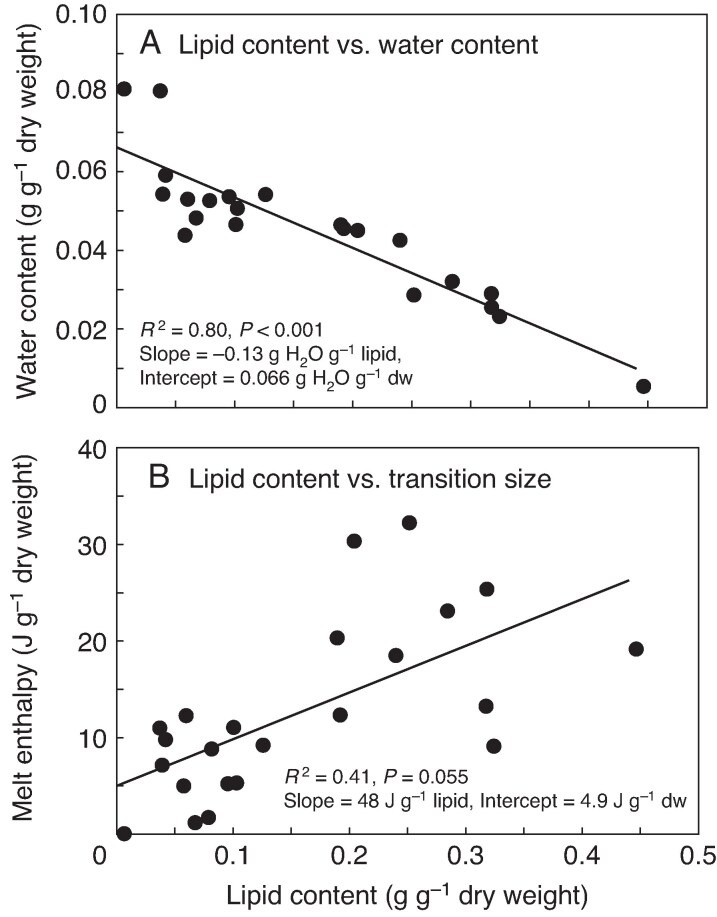
Relationships between lipid content of seeds from Australian rainforest species and (A) water content and (B) total melting enthalpy. Lipid content in (A) uses data from quantitative extractions. The solid line represents the correlation model. Lipid content in (B) averages values from quantitative extractions and predictions from the correlation model with water content in (A). The *y*-axis in (B) is taken from Fig. 3B (*x*-axis) and the solid line represents the correlation model.

We directly determined lipid content in most seeds using quantitative extractions (Table 3); however, this method is cumbersome and costly in terms of labour and seeds. An alternative approach makes use of the apparent linear relationship between lipid content and water content at constant RH and temperature (Vertucci and Roos, 1990; Walters and Hill, 1998). Using this approach, we averaged water content values measured for samples at ambient conditions prior to DSC or lipid extraction as well as those held at controlled relative humidities of 33 and 54 %. As water and lipid contents were highly correlated (*r*^2^ = 0.80, *P* < 0.001, slope = −0.13 g H_2_O g^−1^ lipid) (Fig. 7A), we used this regression model to troubleshoot quantitative extraction data and to fill in lipid content when extractions were not possible (Table 3).

We also sought to use *ΔH*_melt_ as a non-destructive indicator of lipid content. Lipid content, measured by quantitative extraction, was only weakly correlated with *ΔH*_melt_ (Fig. 7B; *P* = 0.055, *r*^2^ = 0.41, slope = 48 J g^−1^ lipid).

Fatty acids were identified by GC from seed lipids for 21 of the 23 species in this study. We observed a preponderance of peaks at retention times in the range where saturated and monounsaturated C16–C20 fatty acids elute (Table 4). GC retention times did not always coincide with our set of standards, which included commonly occurring fatty acids as well as less common isomers that could be purchased, and this may indicate the presence of unidentified isomers; we grouped these isomers with the most common form in lieu of a positive identification.

**Table 4.** Fatty acid proportions in TAGs extracted from seeds of 21 Australian rainforest species. Seeds for all species were collected from subtropical or warm temperate habitats; species in bold text have a distribution extending to the tropics. Cn represents the number of carbons in the fatty acid chain; the degree of saturation is represented by Cn:0 (saturated, SFA), Cn:1 (mono-unsaturated, MUFA) and Cn:2+ (polyunsaturated, PUFA).

Taxon	Caprylic	Myristoleic	Palmitic	Palmitoleic + isomers	Stearic	Oleic + isomers	Linoleic + isomers	Linolenic + isomers	Arachidic	Gadoleic + isomers	Eicosadienoic	Behenic	Total SFAs	Total MUFAs	Total PUFAs
	C8:0	C14:1	C16:0	C16:1	C18:0	C18:1	C18:2	C18:3	C20:0	C20:1	C20:2	C22:0			
***Alphitonia oblata***			0.07		0.07	0.26	0.12	0.46	0.02				0.16	0.26	0.58
***Archirhodomyrtus beckleri***			0.05		0.02	0.09	0.84						0.07	0.09	0.84
***Baloghia inophylla***		0.06	0.18	0.02	0.10	0.25	0.16		0.03		0.15	0.06	0.37	0.32	0.31
***Callicarpa pedunculata***			0.06		0.07	0.14	0.73						0.13	0.14	0.73
***Elaeodendron australe* var*. australe***	0.02		0.16		0.04	0.33	0.45						0.22	0.33	0.45
***Emmenosperma alphitonioides***			0.11		0.09	0.36	0.39	0.04	0.01				0.22	0.36	0.43
***Gynochthodes jasminoides***			0.09		0.05	0.11	0.74	0.01					0.14	0.11	0.75
***Hymenosporum flavum***			0.06		0.01	0.25	0.14			0.53			0.08	0.79	0.14
***Melastoma affine***			0.06		0.02	0.06	0.85		0.01				0.09	0.06	0.85
***Pollia crispata***			0.13		0.03	0.42	0.28	0.10	0.04				0.20	0.42	0.38
***Polyscias murrayi***			0.03		0.03	0.88	0.06						0.06	0.88	0.06
**Average**			**0.09**		**0.05**	**0.29**	**0.43**	**0.12**	**0.02**				**0.16**	**0.34**	**0.50**
**Standard deviation**			**0.05**		**0.03**	**0.22**	**0.29**	**0.17**	**0.01**				**0.09**	**0.26**	**0.26**
*Acalypha capillipes*			0.07	0.05	0.02	0.06	0.16	0.64					0.09	0.11	0.8
*Acradenia euodiiformis*			0.05		0.06	0.40	0.40	0.09					0.10	0.40	0.49
*Capparis anomala*			0.1	0.09	0.08	0.51	0.13	0.08	0.01				0.2	0.59	0.21
*Ceratopetalum apetalum*			0.17	0.02	0.06	0.39	0.35	0.01	0.01				0.23	0.41	0.36
*Denhamia silvestris*			0.14		0.04	0.11	0.69	0.01	0.01				0.19	0.11	0.70
*Ehretia acuminata*			0.07		0.04	0.17	0.72	0.01					0.11	0.17	0.73
*Lenwebbia prominens*			0.09		0.02	0.08	0.80						0.11	0.09	0.80
*Lomandra spicata*			0.08		0.02	0.08	0.82						0.10	0.08	0.82
*Psychotria daphnoides*			0.08		0.04	0.19	0.69						0.12	0.19	0.69
*Rhodamnia maideniana*			0.06		0.02	0.08	0.84						0.08	0.08	0.84
Average			**0.09**	**0.05**	**0.04**	**0.21**	**0.56**	**0.11**	**0.01**				**0.13**	**0.22**	**0.64**
Standard deviation			**0.04**	**0.04**	**0.02**	**0.16**	**0.27**	**0.22**	**0.00**				**0.05**	**0.18**	**0.22**

Fatty acids common to all species were palmitic (C16:0), stearic (C18:0), oleic (C18:1) and linoleic (C18:2), with the bulk of fatty acids for most species consisting of the latter two (oleic and linoleic). High proportions of other fatty acids were found in *Alphitonia oblata* and *Acalypha capillipes* (46 and 64 % linolenic (C18:3), respectively), and *Hymenosporum flavum* (53 % gadoleic, C20:1). On average, seed from species with a distribution that extended to the tropics had slightly higher levels of saturated and monounsaturated fatty acids (SFAs and MUFAs, respectively), and slightly lower levels of polyunsaturated fatty acids (PUFAs) compared with species restricted to subtropical and temperate zones (Table 4); however, these differences were not significant (*P* > 0.3 in each case). Four ‘tropical’ species with low levels of MUFAs and a very high proportion of PUFAs were *Archirhodomyrtus beckleri* (84 % PUFAs), *Callicarpa pedunculata* (73 % PUFAs), *Gynochthodes jasminoides* (74 % PUFAs) and *Melastoma affine* (85 % PUFAs; Table 4).

High levels of SFAs and MUFAs (≥0.61 g fatty acid g^−1^ lipid) were observed in seeds exhibiting the highest temperatures for the largest melting peak (Fig. 2: *Polyscias murrayi*, 24.8 °C; *Baloghia inophylla*, 4.3 °C; *Hymenosporum flavum*, −0.2 °C; *Capparis anomala*, −10.4 °C; and *Ceratopetalum apetalum*, −11.4 °C), though the nature and proportion of different fatty acids varied considerably among species. Species that exhibited a substantial reduction (≥30 %, Table 1) in germination percentage after drying (desiccation-intermediate) had a significantly higher proportion of SFAs + MUFAs (median = 0.61, *n* = 6) than species that were more tolerant of drying but showed a substantial reduction in germination after storage at −20 °C (‘temperature-intermediate’) (median = 0.23, *n* = 10; *P* = 0.008). The proportion of PUFAs in the desiccation-intermediate species (median = 0.395) was significantly lower than in the temperature-intermediate species (median = 0.775, *P* = 0.009).

**Fig. 8.**
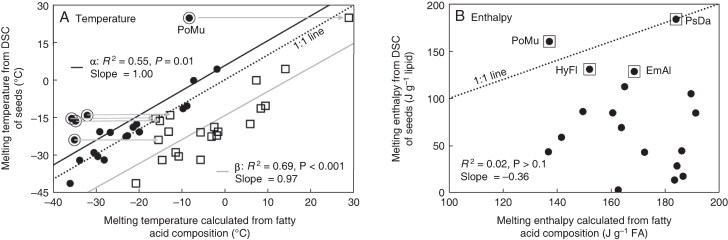
Correspondence of thermal behaviour of lipids predicted from fatty acid composition (*x*-axes) and experimental values of seed tissues measured using DSC (*y*-axes). Experimental data for temperature (A) are from Fig. 3A (*x*-axis) and for enthalpy (B) are from Fig. 5B (*x*-axis). Lines showing a 1:1 relationship are provided. In (A), expected temperature of melting (*T*_melt_) for α (solid circles) and β′ (open squares) crystalline structures are given and both relationships correlate with experimental *T*_melt._(α: slope = 1.00, intercept = 5.6 °C, *r*^2^ = 0.55, *n* = 21, *P* = 0.01; β′: slope = 0.97, intercept = −14.3 °C, *r*^2^ = 0.69, *n* = 21, *P* < 0.001). The five encircled points appear as outliers in the regression with *T*_melt_ expected for α crystals and experimental *T*_melt_ more closely aligned to expected *T*_melt_ of β′ crystals (i.e. open squares are closer to the 1:1 line for that species). In (B) the expected and experimental enthalpies (*ΔH*_melt*_, J g^−1^ lipid) of melting transitions were not correlated (*r*^2^ = 0.10, *n* = 13, *P* > 0.10).

We found a significant correlation between textbook temperatures for α crystalline structures of fatty acids and empirically determined main melting peak temperatures (Fig. 8A; solid circles, *r*^2^ = 0.55, *P* = 0.01, slope = 1.00), though the intercept (5.7 °C) was >0 °C. There were some seed species that appeared as outliers in the relationship, presenting empirical *T*_melt_ values higher than expected for α crystals (encircled points in Fig. 8A). The *T*_melt_ for these seeds was intermediate between expectations for α and β′ crystals (open squares in Fig. 8A). Indeed, regressing empirical *T*_melt_ values with textbook values for β′ crystals resulted in a stronger, almost parallel, correlation (*r*^2^ = 0.69, *P* < 0.001, slope = 0.97), with a significantly lower intercept (−14.3 °C). This analysis demonstrated predictability of DSC-measured *T*_melt_ values based on fatty acid composition, as well as a tendency for seed TAGs in some species (e.g. *Archirhodomyrtus beckleri*, *Polyscias murrayi*, *Baloghia inophylla*, *Melastoma affine* and *Lenwebbia prominens*) to more readily form more stable, regular and compact β′ crystals during our 10 °C min^−1^ cooling/warming protocols. We direct the reader's attention to the thermograms of *Archirhodomyrtus beckleri* following different cooling rates (Fig. 4A), and how the preponderance of the melting peaks at −40 and −18 °C (presumably representing α and β′ crystals, respectively) changed with cooling rate.

In contrast to crystallization behaviour measured by *T*_melt_ (Fig. 8A), there was virtually no relationship between textbook and empirical values of melting enthalpy (Fig. 8B; *r*^2^ = 0.02, *P* > 0.10). As indicated earlier, melting enthalpies (*ΔH*_melt*_, J g^−1^ lipid) of samples with straightforward cooling and warming regimens were usually lower than expected; however, experimental *ΔH*_melt*_ values for *Emmenosperma alphitonioides*, *Hymenosporum flavum, Psychotria daphnoides* and *Polyscias murrayi* were comparable to expected *ΔH*_melt*_ values (boxed points in Fig. 8B).

### Response to storage at −20 °C and alternative temperatures

All species showed high germination after drying (Table 2), including two species (*Elaeodendron australe* var. *australe* and *Emmenosperma alphitonioides*) that had previously shown a substantial reduction in germination when dried at 15 % RH (Table 1). The alternative temperatures selected for storage experiments were warmer than the first crystallization peak (e.g. Fig. 1, peak 1), or cooler than the last crystallization peak (e.g. Fig. 1, peak 3), for all species; one or more of the storage temperatures above −20 °C was also warmer than the final melting transition (e.g. Fig. 1, melt 3) for all species except *Pittosporum multiflorum*, which had a final melting peak at 28 °C (Supplementary Data Fig. S1).

**Fig. 9.**
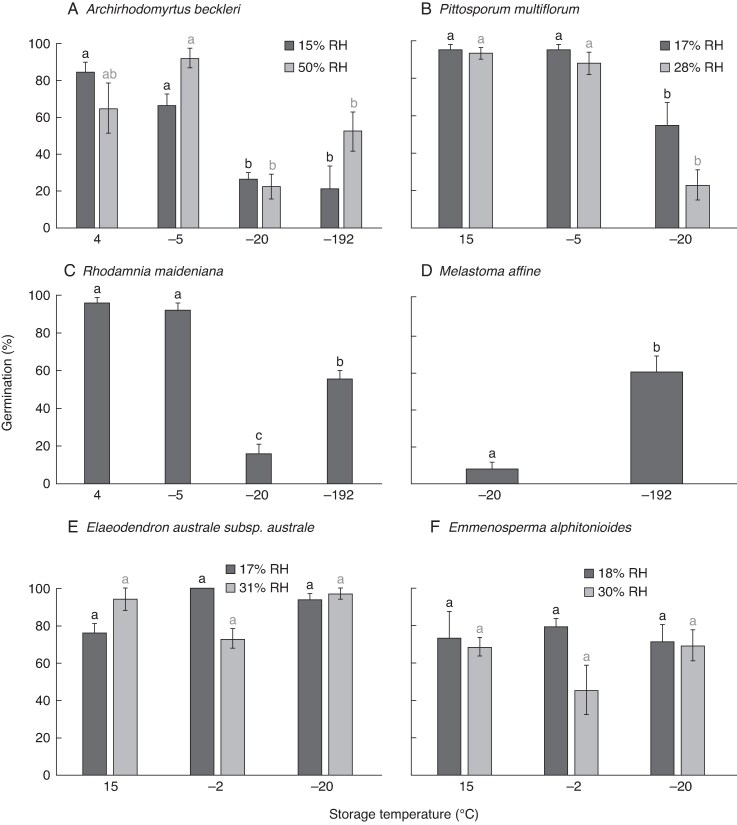
Mean germination (± standard error) of six Australian rainforest species following drying and storage at −20 °C and alternative temperatures chosen to avoid thermal transitions observed during DSC. Each treatment was applied to three to five replicates of 10–20 seeds, depending on seed availability. RH is the equilibrated relative humidity of a seed lot; for charts without a legend, RH = 15 %. Significant differences among treatments are indicated by different letters above the bars; where a significant interaction was found between storage temperature and RH (A, B and E), letters above bars apply to temperature treatments within a single RH treatment and are colour-coded to match the relevant bars. Seeds of *A. beckleri* and *P. multiflorum* were stored for 4 weeks; seeds of *E. alphitonioides* and *E. australe* var. *australe* were stored for 8 weeks; seeds of *M. affine* and *R. maideniana* were stored for 1 year.

A brief period of storage (≤12 months) at −20 °C greatly reduced germination in seeds of *Archirhodomyrtus beckleri*, *Melastoma affine*, *Pittosporum multiflorum* and *Rhodamnia maideniana* compared with storage at alternative temperatures (Fig. 9A–D; Supplementary Data Table S2). For *A. beckleri*, *P. multiflorum* and *R. maideniana*, storage at temperatures *warmer* than transitions observed by DSC (i.e. ≥−5 °C) resulted in the highest post-storage germination percentages (*Melastoma affine* was not tested at warmer temperatures). Storage at a temperature *colder* than transitions observed by DSC (i.e. −192 °C) improved post-storage germination over storage at −20 °C for *A. beckleri*, *R. maideniana* and *M. affine* (Fig. 9A, C, D) but not to the extent of seeds stored at warmer temperatures.

For the *Archirhodomyrtus beckleri* experiment conducted in 2015 (testing three storage temperatures, three moisture contents and two thawing temperatures; Table 2), mean germination ranged from 91 to 98 % following storage at 4 °C compared with 10 to 30 % following storage at −20 or −192 °C (data not displayed). A balanced ANOVA indicated storage temperature had a significant effect on post-storage germination (*P* < 0.001) with no significant interactions among treatments. For the *A. beckleri* experiment conducted in 2022 (testing four storage temperatures and two moisture levels; Fig. 9A), storage temperature again had a significant effect on germination (*P* < 0.001). In this case, however, a significant interaction was also detected among temperature and moisture treatments (*P* = 0.017), with seeds adjusted to 50 % RH having germination slightly (though not significantly) greater after storage at −5 and −192 °C compared with seeds adjusted to 15 % RH (Fig. 9A). Similarly, *Pittosporum multiflorum* showed a significant effect from storage temperature (*P* < 0.001) as well as a significant interaction between storage temperature and moisture level (*P* = 0.045) (Fig. 9B).

In contrast to the above species, germination of *Elaeodendron australe* var. *australe* and *Emmenosperma alphitonioides* remained high following the 2-month exposure to −20 °C, with no significant differences among storage temperatures detected for either species (Fig. 9E, F). A two-way ANOVA detected a significant interaction between storage temperature and moisture level for *E. australe* var. *australe* (*P* = 0.015), with germination after storage at −2 °C being slightly (though not significantly) greater for seeds equilibrated to 17 % RH compared with 31 % RH (Fig. 9E).

## DISCUSSION

The longevity of seeds in storage is of great importance to the conservation of both wild and crop species, determining how long a seed collection will remain useful for regenerating whole plants. This is particularly important to species on the verge of extinction, for which recovery may depend entirely on seeds held in a gene bank. Studies on seed longevity have found wide variation in the trait among species (Walters *et al*., 2005; Probert *et al*., 2009; Mondoni *et al*., 2011; Merritt *et al*., 2014), with real-time half-lives ranging from less than a decade to several centuries in cool (5 °C) and cold (−18 °C) storage, respectively (Colville and Pritchard, 2019). Factors affecting longevity in storage for orthodox seeds have been the subject of considerable research, with both intrinsic factors (antioxidant compounds, stress proteins, non-reducing sugars and DNA repair mechanisms) and extrinsic factors (e.g. nutrient and water availability during seed development) found to contribute to seed lifespan (Zinsmeister *et al*., 2020). In contrast, the longevity of intermediate seeds in storage is relatively understudied.

Here we investigated seeds from 23 species native to the rainforests of Eastern Australia that had been found to be short-lived under standard gene-banking conditions (Sommerville *et al*., 2021, Table 1). Seeds of these species were relatively tolerant of drying (so not recalcitrant *sensu* Roberts, 1973), but some exhibited a reduction in germination after drying to 15 % RH and most exhibited faster-than-expected viability loss following subsequent exposure to −20 °C, suggesting temperature-sensitive intermediate storage behaviour (Hong and Ellis, 1996). As the thermal behaviours of storage lipids have been hypothesized to play a role in such behaviour (e.g. Vertucci, 1992; Crane *et al*., 2003; Mira *et al*., 2019), we applied DSC and fatty acid analysis to (1) identify thermal events (i.e. crystallization and melting) that occur in the dry seeds near −20 °C; (2) assess the contribution of seed oils to the observed thermal behaviour; (3) test whether tropical oils (i.e. those with a high proportion of saturated or monounsaturated fatty acids) could be implicated in the unexpected viability loss; and (4) test whether storage at temperatures outside the range of thermal events could improve longevity.

DSC thermograms revealed substantial endothermic and exothermic activity near −20 °C in most of the seeds in this study set (Figs 1, 2, 4 and 5). These transitions have been definitively attributed to TAGs based on the high correspondence of melting temperatures *in vivo* with TAGs extracted from seeds (Figs 5 and 6A), as was reported for *Citrus grandis* and *C. inodora* (Hor *et al*., 2005; Hamilton *et al*., 2009). Moreover, the melting temperatures observed in these seeds were consistent with expected temperatures based on fatty acid composition (Fig. 8A). This result provided additional confirmation that the DSC transitions observed in seed samples could be attributed to TAGs.

The much greater size of thermal transitions on melting compared with those observed on cooling suggested substantial crystallization of lipids occurred during the *warming* phase of the protocol used in this study. In particular, the large and sharp size of transitions within *Baloghia inophylla* seeds on warming (Fig. 2D, dashed curve), despite negligible activity observed on cooling (Table 3; Fig. 3B, encircled point at 32 J g^−1^ on *x*-axis), suggested the rapid formation of consistently-shaped crystals during warming from −150 to −20 °C at 10 °C min^−1^.

The shapes of DSC warming thermograms varied greatly among species in this study, even for those with a similar fatty acid composition. For example, among seeds containing high levels of linoleic acid, some had prominent recrystallization events near −30 °C (i.e. those with the highest linoleic acid content) and others had relatively small and broad transitions with multiple ‘peaks’ that were difficult to interpret. After slower cooling or suitable annealing, recrystallization events during warming were reduced or eliminated and the warmest peak became sharper and more prominent for several species (Fig. 4, especially *Callicarpa pedunculata*), indicating that crystallization and melting can occur concurrently during warming depending on the rate of cooling/warming used.

The complex thermal behaviours observed between −25 and 0 °C from warming thermograms of most of the study species (Fig. 2) likely reflect heterogeneity in the speed of TAG crystal growth as well as the molecular structure of the crystals. Large variation in the shape and size of melting endotherms based on cooling rate indicated that the kinetics of crystallization were complex, possibly including discrete nucleation events (i.e. alignment of a small number of TAG molecules into a crystalline pattern) and slow crystal growth and rearrangement. Initial nucleation appeared inhibited, based on substantial supercooling of TAGs in many of the seeds (Fig. 3A; points to the right of the 1:1 line indicate *T*_crys_ < *T*_melt_). The tiny exothermic events observed upon cooling (Fig. 1) and small *ΔH*_crys_ relative to *ΔH*_melt_ (Fig. 3B) indicated that only a modicum of crystal growth occurred during the cooling phase of the protocol used. Crystallization events during warming, observed in many seeds near −80 °C, suggested that some fraction of quasi-aligned TAG molecules crystallized into more organized structures (Fig. 1; recrystallization following melting peak 1). Another recrystallization event occurred in some seeds between −40 and −5 °C (Figs 1, 2 and 4), indicating the additional recruitment of molecules into crystalline structures during warming.

Slow crystallization of TAGs was not restricted to the seed system. Indeed, extracted TAGs showed remarkable resistance to crystallization, as evidenced by the extremely low *ΔH*_melt*_ of extracted lipids (average = 36 J g^−1^ lipid) compared with *ΔH*_melt*_in vivo (average = 79 J g^−1^ lipid), and compared with values obtained following slow cooling (Fasina *et al*., 2008) or textbook values for individual fatty acids (∼160 J g^−1^ lipid depending on fatty acid chain length, crystal structure and method of determination; Fig. 8B; Small, 1986; Hernqvist, 1990; Zeberg-Mikkelson and Stenby, 1999; Cedeño *et al*., 2001). In fact, procedures to obtain accurate textbook values for *ΔH*_melt*_ of extracted lipids are complex and involve treatments to nucleate and grow crystals with a known structure, such as our annealing experiments with seeds of *Callicarpa pedunculata* (Fig. 4D). The relatively poor correlation between melting enthalpy (*ΔH*_melt_) and TAG content (Fig. 7B) indicated that the rate of TAG crystal growth varied among species. In seeds of a few species, *ΔH*_melt*_in vivo approached the textbook value (160 J g^−1^ lipid), suggesting that crystallization was almost complete (i.e. *Emmenosperma alphitonioides*, *Hymenosporum flavum*, *Polyscias murrayi* and *Psychotria daphnoides*). Except for *P. murrayi*, these species had low TAG content (Figs 3B and 6B), which may suggest that the surface area:volume ratio of lipid bodies or the presence/absence of oleosins in lipid–body membranes influence TAG crystallization rates.

TAGs crystallize into three main forms (α, β′ and β), each of which has a different crystalline structure and a different melting point (Hernqvist, 1990). These polymorphic structures add even greater complexity to the kinetics of crystallization. Lower-density, higher-energy α crystals (Fig. 1, melting peak 2), which have been called ‘meta-stable’ (Mira *et al*., 2019), convert to higher-density, lower-energy β′ (Fig. 1, melting peak 3) and β forms (not shown) over time at temperatures slightly below and encompassing the α melting endotherm (Small, 1986; Hernqvist, 1990; Zeberg-Mikkelson and Stenby, 1999). Conversion of α → β′ crystals was demonstrated here by greater prominence of the higher temperature peak (β′) in the slow cooling and annealing experiments (Fig. 4), as seen in previous studies of seeds (e.g. *Brassica* species; Mira *et al*., 2019) and fern spores (Ballesteros *et al*., 2019) stored at −20 °C. The annealing experiment using *Callicarpa pedunculata* (Fig. 4D) illustrated how subzero temperature fluctuations to encourage nucleation, combined with low temperature storage to encourage crystal growth, can lead to a preponderance of β′ crystals.

The rate that disorganized α crystals convert to denser, more organized β′ and β forms appears to be species-dependent. For example, while *T*_melt_ for most of the species identified α crystals as the prominent form following 10 °C min^−1^ cooling (Fig. 8A), *T*_melt_ for seeds of *Archirhodomyrtus beckleri*, *Lenwebbia prominens*, *Melastoma affine*, *Polyscias murrayi* and *Rhodamnia maideniana* indicated that α → β′ conversions were occurring faster in these species (Fig. 8A, encircled points). Note that germination of three of these species – *A. beckleri*, *M. affine* and *R. maideniana* – showed extreme and rapid sensitivity to −20 °C on re-testing (Fig. 9). The TAGs within seeds of these species were relatively pure, with either oleic or linoleic acid comprising >80 % of the fatty acid composition (Table 4). In contrast, the α → β′ conversion seemed slow in *Acalypha capillipes* and *Callicarpa pedunculata* seeds, in which thermogram shape was similar for seeds cooled at either 1 or 10 °C min^−1^ (Fig. 4).

The tropical distribution of 11 of the species in this study set might suggest that their lipids would be rich in tropical oils, i.e. long-chain SFAs and MUFAs (Linder, 2000; Salas *et al*., 2009). The average proportions of tropical oils in this subset of species, however, were not significantly different from the proportions found in species restricted to subtropical and temperate zones (Table 4). High levels of tropical oils, however, corresponded with high *T*_melt_ and low survival during freezer storage. Of the five species with the highest proportions of SFAs + MUFAs, four showed a substantial reduction in germination after drying and freezing (Table 1), consistent with the notion that seeds accumulating high levels of tropical oils may not be suitable for freezer storage (Crane *et al*., 2003, 2006). The species that did not fit this pattern (*Hymenosporum flavum*) produced seeds that survived both drying (91 % germination) and cold storage for around 2 years (73 % germination) (Table 1). Seeds of *H. flavum* had a lower TAG content (0.06 g g^−1^ dw) compared with the other four species (0.19–0.25 g g^−1^ dw, Table 3) and this was the only species with a high proportion (53 %) of monounsaturated gadoleic (20:1) acid (Table 4), suggesting that both the quantity of TAGs and the relative proportions of constituent fatty acids influence a species’ response to drying and cold storage.

Many of the species considered here contained high proportions of PUFAs, rather than tropical oils. Linoleic acid (18:2), for example, was unusually prevalent in ten species (0.69–0.85 g g^−1^ lipid) while linolenic acid (18:3) was prevalent in *Acalypha capillipes* (0.64 g g^−1^ lipid; Table 4). Species with high linoleic or linolenic acid can be grouped into those that survived drying but were severely damaged by storage at −20 °C (i.e. temperature-intermediate: *Acalypha capillipes*, *Archirhodomyrtus beckleri*, *Callicarpa pedunculata*, *Denhamia sylvestris*, *Gynochthodes jasminoides*, *Lenwebbia prominens*, *Melastoma affine* and *Rhodamnia maideniana*; Table 1) and those that showed substantially reduced germination after drying but then little further change in storage (i.e. desiccation-intermediate: *Ehretia acuminata*, *Lomandra spicata* and *Psychotria daphnoides*; Table 1). The desiccation-intermediate seeds tended to have low TAG content (≤0.10 g lipid g^−1^ dw) and, for two species, moderate amounts of SFAs + MUFAs (*Ehretia acuminata* 28 % and *Psychotria daphnoides* 31 %), giving further support to the notion that both the quantity and composition of storage lipids influence a species’ response to drying and storage.

Analysis of the 16 species that showed a reduction in germination of ≥30 % after drying or storage (Table 1) suggested that seeds high in SFAs + MUFAs tend to be more sensitive to drying while those high in PUFAs tend to be relatively tolerant of drying but sensitive to freezing. This pattern was not evident, however, in relatives of *Brassica* (Mira *et al*., 2019) or tropical *Cuphea* species (Crane *et al*., 2003). Examination of species from a broader range of families would help to resolve whether the pattern is unique to this study or more widely applicable. Investigation of factors that moderate the impact of lipid transitions on cellular structure, such as the relative abundance of oleosins in lipid-body membranes (Leprince *et al*., 1998), may aid in understanding different responses to drying and freezing among species with similar fatty acid composition.

Examining the thermal properties of TAGs presents a novel perspective of the chemical and physical stabilization needed for seed longevity (Walters *et al*., 2010; Walters, 2015; Ballesteros *et al*., 2020). Lipids have long been implicated in seed ageing and poor longevity. Historically, studies have focused on orthodox seeds and pollen and explored the chemical instability of PUFAs, implicating them in oxidative reactions believed to cause ageing (Priestley, 1986; Hoekstra, 2005; Wiebach *et al*., 2020; Zinsmeister *et al*., 2020). These studies arose from long-standing anecdotal relationships between ageing rates and PUFAs as well as poor longevity in oily seeds. The latter hypothesis was debunked for orthodox seeds (Priestley, 1986; Walters *et al*., 2005, Probert *et al*., 2009) but has not really been tested for seeds in the intermediate storage category, which may involve structural attributes of lipids rather than chemical reactivity.

Here, we focused on TAG structural stability within the temperature range near −20 °C. We demonstrated that TAGs crystallize slowly and incompletely when cooled quickly or stored at temperatures between *T*_crys_ and *T*_melt_, generating a population of thermodynamically unstable structures in lipid bodies. The structures may be kinetically stabilized at sufficiently cold conditions (such as below −80 °C). However, at warmer temperatures <*T*_melt_ (such as the standard −18 ± 3 °C used for seed banking), crystals grow and realign into denser and denser structures, the greater packing efficiency causing the volume of lipid domains to shrink. An extant hypothesis is that ever-shrinking lipid domains embedded in glassy domains of the cytoplasm of dry seeds will create larger pore spaces and destabilize the glassy matrix (Walters, 2015). The time and spatial scales involved, as well as the temperature dependencies of motion, may differ considerably in different parts of the cell, resulting in complex kinetics in which the detrimental effect of low-temperature storage may be immediate or may be delayed for some years (Ashmore *et al*., 2008; Ballesteros *et al*., 2019; Chau *et al*., 2019; Fleming *et al*., 2019; Mira *et al*., 2019; Sommerville *et al*., 2023).

Two practical questions addressed in this study were whether exposure to −20 °C would be more damaging to seeds than other temperatures, and whether storage at temperatures that avoided TAG transitions would improve longevity. Extreme sensitivity to −20 °C was confirmed for *Archirhodomyrtus beckleri*, *Melastoma affine*, *Rhodamnia maideniana* and *Pittosporum multiflorum* (Fig. 9A–D). For three of these species (*A. beckleri*, *R. maideniana* and *P. multiflorum*), short-term storage at temperatures warmer than −20 °C greatly improved post-storage germination (Fig. 9A–C). For *A. beckleri* and *R. maideniana*, the warmer storage temperatures (−5 and 4 °C) avoided all crystallization and melting transitions detected by DSC (Fig. 4C). For *P. multiflorum*, the warmer storage temperatures (−5 and 15 °C) avoided all detected crystallization transitions but fell between *T*_crys_ and a final melting event above room temperature (28 °C; Supplementary Data Fig. S1). Given that the temperature used to thaw *P. multiflorum* seeds (∼23 °C) was lower than the warmest melting peak for the species, it is possible that incomplete melting of crystallized lipids contributed to the reduction in germination following storage at −20 °C (Crane *et al*., 2003). In addition, while storage at −5 and 15 °C was more beneficial than storage at −20 °C for *P. multiflorum* in the short term, it is possible that slow crystallization of lipids may occur at those temperatures over a longer time frame. For all three species, longer-term storage at temperatures above zero may also increase the risk of oxidative reactions associated with seed ageing.

In contrast to the four species above, 2 month's storage at −20 °C had no significant impact on the germination of *Elaeodendron australe* var. *australe* or *Emmenosperma alphitonioides* compared with storage at other temperatures (Fig. 9E, F). For both species, germination after drying of the garden-collected seeds was considerably better than in earlier studies on wild-collected seed (≥82 vs 46 %, respectively; Tables 1 and 2). This improvement in seed quality likely contributed to better survival in storage in the short term; however, the poor longevity in storage exhibited by the wild collections of both species (Table 1; Sommerville *et al*., 2021) suggests a decline in viability over time could be expected. The longer time-frame for decline in these species compared with those that showed a substantial decline in just 1 month (*A. beckleri* and *P. multiflorum*) illustrates the practical consequence of variation in crystallization kinetics.

Storage at liquid nitrogen temperatures could be expected to improve longevity in these short-lived species by avoiding TAG transitions. Depending on the speed of crystallization during cooling, liquid nitrogen temperatures should limit crystal growth and conversions to other crystal forms during storage. For three of the four species most sensitive to −20 °C (Fig. 9A, C, D), germination following storage at −192 °C was greater than following storage at −20 °C but was still significantly less than germination after storage at warmer temperatures for two species (Fig. 9A, C). The cooling and warming protocols used could be a contributing factor: rates should be optimized to avoid β′ conversions (which are fast in these species) and mechanical damage from cracking (Vertucci, 1989). Moisture levels should also be optimized to avoid the detrimental effects of over-drying intermediate-type seeds (e.g. Hong and Ellis, 1996; Sacandé *et al*., 2000; Hor *et al*., 2005; Walters, 2015; Ballesteros and Pence, 2017). The importance of correctly adjusted moisture content was demonstrated here by the significant interaction between temperature and moisture for three of the four species in which two moisture levels were studied (Fig. 9A, B, E).

Several studies have highlighted the importance of provenance to variation in seed longevity, with species from warm, dry climates or low altitudes tending to be longer-lived than those from cool, moist climates or high altitudes (Walters *et al*., 2005; Probert *et al*., 2009; Mondoni *et al*., 2011; Sommerville *et al*., 2023). Taxonomy and growth form (Merritt *et al*., 2014) and seed morphology (Probert *et al*., 2009) have also been shown to have an influence on longevity. Here, we have shown a relationship between poor longevity in intermediate rainforest seeds and the thermal behaviour of seed storage lipids and we have demonstrated that longevity can be improved by storage at temperatures that avoid or minimize structural changes to those lipids. DSC provides a means to rapidly detect phase changes in lipids that may confound successful seed banking at −20 °C and a means for identifying more appropriate storage temperatures. The technique is useful as an initial screen of storage behaviour, especially when seed supply is limited or seeds are difficult to germinate.

### Conclusions

We examined a trend of poor seed longevity in rainforest species from eastern Australia and found that lipid phase transitions near −20 °C were apparent in all seeds that died unexpectedly quickly. Limiting lipid structural changes might be possible using cryopreservation if cooling and warming rates and hydration status can be optimized through further empirical study. In the interim, the longevity of seeds short-lived at −20 °C may be improved by storing dry seeds at temperatures warmer than lipid transitions (i.e. above −20 °C) as guided by DSC thermograms. Further experiments are required to determine how long this approach will remain effective.

## Supplementary Material

mcaf181_Supplementary_Data

## Data Availability

Data used for regression analyses and to prepare Figs 3 and 6–9 are available in Tables 3 and 4 and Supplementary Data Tables S1, S2. Raw data generated by differential scanning calorimetry and used to create Figs 1, 2, 4 and 5 and Supplementary Data Fig. S1 may be obtained by contacting the lead author.

## SUPPLEMENTARY DATA

Supplementary data are available at *Annals of Botany* online and consist of the following. Table S1: enthalpy and temperature of melting transitions for *in vivo* and extracted seed lipids. Table S2: raw data for post-storage germination of six Australian rainforest species. Figure S1: cooling and warming thermograms for dry seeds of *Pittosporum multiflorum*.
