# Supplementary material for: Thermal behaviour of lipids in short-lived seeds of Australian rainforest species
Source: Ann Bot. 2025 Aug 6;136(7):1547–64. doi: 10.1093/aob/mcaf181 (PMC12718007; doi:10.1093/aob/mcaf181)

Supplementary Figure 1

Cooling (lower) and warming (upper) thermograms for dry seeds of *Pittosporum multiflorum*. Seed samples were cooled from 30 to -150°C and warmed from -150 to 50°C at a rate of 10°C per minute (transitions between -50 and 50°C are shown here; no transitions occurred outside that range). Crys 1 and 2 represent crystallisation transitions upon cooling. Melt 1, 2 & 3 represent melting transitions upon warming. Note the final melting peak is at 28.7°C.

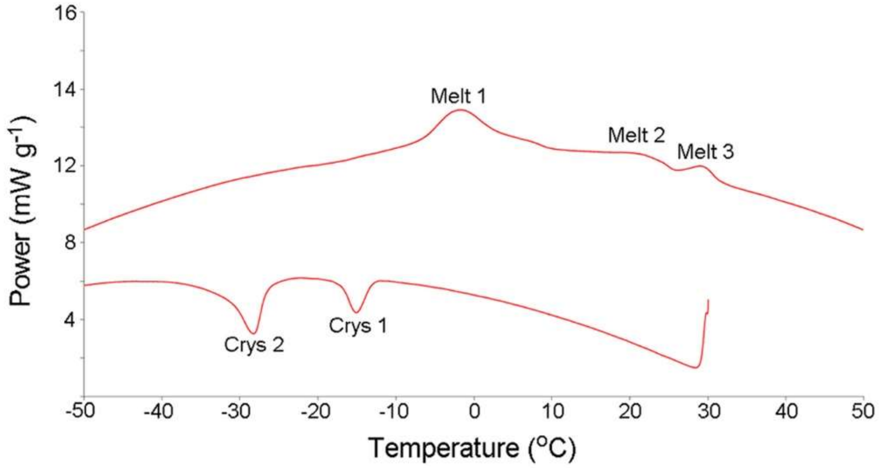

Supplement: mcaf181_Supplementary_Data [file mcaf181_supplementary_data.zip › Supplementary Figure 1.pdf]
