# Supplementary material for: Thermal behaviour of lipids in short-lived seeds of Australian rainforest species
Source: Ann Bot. 2025 Aug 6;136(7):1547–64. doi: 10.1093/aob/mcaf181 (PMC12718007; doi:10.1093/aob/mcaf181)
Supplement: mcaf181_Supplementary_Data [file mcaf181_supplementary_data.zip › Supplementary Table 1.pdf]

Supplementary Table 1

Enthalpy (ΔH_{melt}) and temperature (T_{melt}) of melting transitions for in vivo and extracted seed lipids of 23 Australian rainforest species. Values as determined by differential scanning calorimetry in which samples were cooled from 20 to -150°C, then warmed from -150 to 50°C, at a rate of 10°C min^{-1}.

	**ΔH_{melt} (total)** (J g^{-1} lipid)		**T_{melt} (largest peak)** (°C)		**T_{melt} (warmest peak)** (°C)	
**Taxon**	**in vivo**	**extracted**	**in vivo**	**extracted**	**in vivo**	**extracted**
Acalypha capillipes	42.8	32.9	-41.4	-39.13	-14.0	-23.3
Acradenia euodiiformis	82.2	59.7	-17.6	-22.13	3.9	-7.47
Alphitonia oblata	43.6	16.7	-20.7	-32.13	0.2	-28.05
Archirhodomyrtus beckleri	75.3	46.4	-15.4	-42.97	-15.4	-17.47
Baloghia inophylla	112.8	43.3	4.3	1.87	4.3	1.87
Callicarpa pedunculata	16.1	28.2	-30.6	-32.13	-28.2	-32.13
Capparis anomala	88.2	52.7	-10.4	-12.47	7.1	16.87
Ceratopetalum apetalum	73.3	23.2	-18.1	-19.97	5.5	-6.13
Denhamia silvestris	33.0	8.9	-22.2	-21.63	-11.6	-21.63
Ehretia acuminata	11.8	31.6	-32.0	-34.65	-32.0	0.2
Elaeodendron australe subsp. *australe*	44.8	20.4	-18.9	-19.78	-6.2	-19.78
Emmenosperma alphitonioides	134.9	45.7	-20.7	-24.47	5.4	-6.35
Gynochthodes jasminoides	41.4	45.0	-37.2	-35.3	-12.1	0.53
Hymenosporum flavum	156.3	5.9	-0.2	8.03	-0.2	8.03
Lenwebbia prominens	86.7	10.0	-14.1	-16.8	-14.1	-16.8
Lomandra spicata	76.4	nd	-32.1	nd	-14.1	nd
Melastoma affine	100.1	39.8	-16.4	-41.47	-16.4	-16.97
Pittosporum multiflorum	97.4	nd	-1.6	nd	28.7	nd
Pollia crispata	3.6	nd	-22.7	nd	-13.0	nd
Polyscias murrayi	165.4	96.0	24.8	20.785	24.8	20.785
Psychotria daphnoides	166.4	22.9	-29.0	-31.97	-16.5	-31.97
Rhodamnia maideniana	94.4	nd	-24.0	nd	-14.8	nd
Syzygium anisatum	84.3	49.4	-18.6	-16.47	9.3	1.7
