# Supplementary material for: Thermal behaviour of lipids in short-lived seeds of Australian rainforest species
Source: Ann Bot. 2025 Aug 6;136(7):1547–64. doi: 10.1093/aob/mcaf181 (PMC12718007; doi:10.1093/aob/mcaf181)
Supplement: mcaf181_Supplementary_Data [file mcaf181_supplementary_data.zip › Supplementary Table 2.pdf]

**Supplementary Table 2**

Raw data for post-storage germination of six Australian rainforest species. Seeds dried to equilibration with ~15, 30 or 50% relative humidity (RH) and stored for 1 to 12 months at the temperatures indicated. Seeds were thawed in a water bath (WB) at 40°C or at room temperature (RT, ~23°C). No. sown = number of viable seeds sown (excluding empty seeds); No. germ. = number of seeds germinating of viable seeds sown; Germ (%) is the percentage of viable seeds germinating; Avg. germ (%) is the average germination for a given set of conditions.

| Species | Storage temp. (°C) | RH (%) | Thawing temp. | No. sown | No. germ. | Germ (%) | Avg. Germ (%) |
|---|---|---|---|---|---|---|---|
| Archirhodomyrtus beckleri 2015 | 4 | 15 | WB | 16 | 14 | 88 | |
| Archirhodomyrtus beckleri 2015 | 4 | 15 | WB | 18 | 18 | 100 | |
| Archirhodomyrtus beckleri 2015 | 4 | 15 | WB | 18 | 16 | 89 | |
| Archirhodomyrtus beckleri 2015 | 4 | 15 | WB | 17 | 15 | 88 | |
| Archirhodomyrtus beckleri 2015 | 4 | 15 | WB | 15 | 15 | 100 | 93 |
| Archirhodomyrtus beckleri 2015 | -20 | 15 | WB | 13 | 3 | 23 | |
| Archirhodomyrtus beckleri 2015 | -20 | 15 | WB | 19 | 3 | 16 | |
| Archirhodomyrtus beckleri 2015 | -20 | 15 | WB | 19 | 3 | 16 | |
| Archirhodomyrtus beckleri 2015 | -20 | 15 | WB | 17 | 2 | 12 | |
| Archirhodomyrtus beckleri 2015 | -20 | 15 | WB | 20 | 2 | 10 | 15 |
| Archirhodomyrtus beckleri 2015 | -192 | 15 | WB | 18 | 5 | 28 | |
| Archirhodomyrtus beckleri 2015 | -192 | 15 | WB | 18 | 1 | 6 | |
| Archirhodomyrtus beckleri 2015 | -192 | 15 | WB | 18 | 4 | 22 | |
| Archirhodomyrtus beckleri 2015 | -192 | 15 | WB | 18 | 1 | 6 | |
| Archirhodomyrtus beckleri 2015 | -192 | 15 | WB | 17 | 0 | 0 | 12 |
| Archirhodomyrtus beckleri 2015 | 4 | 30 | WB | 20 | 19 | 95 | |
| Archirhodomyrtus beckleri 2015 | 4 | 30 | WB | 15 | 13 | 87 | |
| Archirhodomyrtus beckleri 2015 | 4 | 30 | WB | 18 | 18 | 100 | |
| Archirhodomyrtus beckleri 2015 | 4 | 30 | WB | 19 | 16 | 84 | |
| Archirhodomyrtus beckleri 2015 | 4 | 30 | WB | 18 | 16 | 89 | 91 |
| Archirhodomyrtus beckleri 2015 | -20 | 30 | WB | 14 | 4 | 29 | |
| Archirhodomyrtus beckleri 2015 | -20 | 30 | WB | 14 | 0 | 0 | |
| Archirhodomyrtus beckleri 2015 | -20 | 30 | WB | 19 | 2 | 11 | |
| Archirhodomyrtus beckleri 2015 | -20 | 30 | WB | 18 | 4 | 22 | |
| Archirhodomyrtus beckleri 2015 | -20 | 30 | WB | 17 | 5 | 29 | 18 |
| Archirhodomyrtus beckleri 2015 | -192 | 30 | WB | 17 | 3 | 18 | |
| Archirhodomyrtus beckleri 2015 | -192 | 30 | WB | 15 | 5 | 33 | |
| Archirhodomyrtus beckleri 2015 | -192 | 30 | WB | 20 | 7 | 35 | |
| Archirhodomyrtus beckleri 2015 | -192 | 30 | WB | 19 | 4 | 21 | |
| Archirhodomyrtus beckleri 2015 | -192 | 30 | WB | 16 | 2 | 13 | 24 |
| Archirhodomyrtus beckleri 2015 | 4 | 50 | WB | 17 | 16 | 94 | |
| Archirhodomyrtus beckleri 2015 | 4 | 50 | WB | 17 | 17 | 100 | |
| Archirhodomyrtus beckleri 2015 | 4 | 50 | WB | 18 | 16 | 89 | |
| Archirhodomyrtus beckleri 2015 | 4 | 50 | WB | 16 | 14 | 88 | |
| Archirhodomyrtus beckleri 2015 | 4 | 50 | WB | 19 | 19 | 100 | 94 |
| Archirhodomyrtus beckleri 2015 | -20 | 50 | WB | 16 | 0 | 0 | |
| Archirhodomyrtus beckleri 2015 | -20 | 50 | WB | 16 | 3 | 19 | |
| Archirhodomyrtus beckleri 2015 | -20 | 50 | WB | 19 | 3 | 16 | |

| Species | Storage temp. (°C) | RH (%) | Thawing temp. | No. sown | No. germ. | Germ (%) | Avg. Germ (%) |
|---|---|---|---|---|---|---|---|
| Archirhodomyrtus beckleri 2015 | -20 | 50 | WB | 18 | 1 | 6 | |
| Archirhodomyrtus beckleri 2015 | -20 | 50 | WB | 17 | 2 | 12 | 10 |
| Archirhodomyrtus beckleri 2015 | -192 | 50 | WB | 17 | 3 | 18 | |
| Archirhodomyrtus beckleri 2015 | -192 | 50 | WB | 18 | 9 | 50 | |
| Archirhodomyrtus beckleri 2015 | -192 | 50 | WB | 17 | 7 | 41 | |
| Archirhodomyrtus beckleri 2015 | -192 | 50 | WB | 18 | 5 | 28 | |
| Archirhodomyrtus beckleri 2015 | -192 | 50 | WB | 16 | 2 | 13 | 30 |
| Archirhodomyrtus beckleri 2015 | 4 | 15 | RT | 17 | 16 | 94 | |
| Archirhodomyrtus beckleri 2015 | 4 | 15 | RT | 19 | 17 | 89 | |
| Archirhodomyrtus beckleri 2015 | 4 | 15 | RT | 18 | 18 | 100 | |
| Archirhodomyrtus beckleri 2015 | 4 | 15 | RT | 16 | 16 | 100 | |
| Archirhodomyrtus beckleri 2015 | 4 | 15 | RT | 18 | 17 | 94 | 96 |
| Archirhodomyrtus beckleri 2015 | -20 | 15 | RT | 18 | 6 | 33 | |
| Archirhodomyrtus beckleri 2015 | -20 | 15 | RT | 18 | 3 | 17 | |
| Archirhodomyrtus beckleri 2015 | -20 | 15 | RT | 14 | 2 | 14 | |
| Archirhodomyrtus beckleri 2015 | -20 | 15 | RT | 15 | 5 | 33 | |
| Archirhodomyrtus beckleri 2015 | -20 | 15 | RT | 15 | 3 | 20 | 24 |
| Archirhodomyrtus beckleri 2015 | -192 | 15 | RT | 16 | 3 | 19 | |
| Archirhodomyrtus beckleri 2015 | -192 | 15 | RT | 18 | 1 | 6 | |
| Archirhodomyrtus beckleri 2015 | -192 | 15 | RT | 17 | 0 | 0 | |
| Archirhodomyrtus beckleri 2015 | -192 | 15 | RT | 16 | 2 | 13 | |
| Archirhodomyrtus beckleri 2015 | -192 | 15 | RT | 18 | 4 | 22 | 12 |
| Archirhodomyrtus beckleri 2015 | 4 | 30 | RT | 17 | 17 | 100 | |
| Archirhodomyrtus beckleri 2015 | 4 | 30 | RT | 17 | 17 | 100 | |
| Archirhodomyrtus beckleri 2015 | 4 | 30 | RT | 19 | 19 | 100 | |
| Archirhodomyrtus beckleri 2015 | 4 | 30 | RT | 17 | 15 | 88 | |
| Archirhodomyrtus beckleri 2015 | 4 | 30 | RT | 20 | 20 | 100 | 98 |
| Archirhodomyrtus beckleri 2015 | -20 | 30 | RT | 17 | 2 | 12 | |
| Archirhodomyrtus beckleri 2015 | -20 | 30 | RT | 17 | 3 | 18 | |
| Archirhodomyrtus beckleri 2015 | -20 | 30 | RT | 14 | 1 | 7 | |
| Archirhodomyrtus beckleri 2015 | -20 | 30 | RT | 17 | 2 | 12 | |
| Archirhodomyrtus beckleri 2015 | -20 | 30 | RT | 16 | 2 | 13 | 12 |
| Archirhodomyrtus beckleri 2015 | -192 | 30 | RT | 19 | 4 | 21 | |
| Archirhodomyrtus beckleri 2015 | -192 | 30 | RT | 18 | 2 | 11 | |
| Archirhodomyrtus beckleri 2015 | -192 | 30 | RT | 18 | 7 | 39 | |
| Archirhodomyrtus beckleri 2015 | -192 | 30 | RT | 17 | 0 | 0 | |
| Archirhodomyrtus beckleri 2015 | -192 | 30 | RT | 19 | 8 | 42 | 23 |
| Archirhodomyrtus beckleri 2015 | 4 | 50 | RT | 20 | 20 | 100 | |
| Archirhodomyrtus beckleri 2015 | 4 | 50 | RT | 16 | 13 | 81 | |
| Archirhodomyrtus beckleri 2015 | 4 | 50 | RT | 17 | 15 | 88 | |
| Archirhodomyrtus beckleri 2015 | 4 | 50 | RT | 17 | 16 | 94 | |
| Archirhodomyrtus beckleri 2015 | 4 | 50 | RT | 17 | 17 | 100 | 93 |
| Archirhodomyrtus beckleri 2015 | -20 | 50 | RT | 19 | 3 | 16 | |
| Archirhodomyrtus beckleri 2015 | -20 | 50 | RT | 16 | 1 | 6 | |
| Archirhodomyrtus beckleri 2015 | -20 | 50 | RT | 17 | 2 | 12 | |

| Species | Storage temp. (°C) | RH (%) | Thawing temp. | No. sown | No. germ. | Germ (%) | Avg. Germ (%) |
|---|---|---|---|---|---|---|---|
| Archirhodomyrtus beckleri 2015 | -20 | 50 | RT | 17 | 1 | 6 | |
| Archirhodomyrtus beckleri 2015 | -20 | 50 | RT | 20 | 3 | 15 | 11 |
| Archirhodomyrtus beckleri 2015 | -192 | 50 | RT | 17 | 3 | 18 | |
| Archirhodomyrtus beckleri 2015 | -192 | 50 | RT | 17 | 10 | 59 | |
| Archirhodomyrtus beckleri 2015 | -192 | 50 | RT | 18 | 6 | 33 | |
| Archirhodomyrtus beckleri 2015 | -192 | 50 | RT | 18 | 4 | 22 | |
| Archirhodomyrtus beckleri 2015 | -192 | 50 | RT | 17 | 5 | 29 | 32 |
| | | | | | | | |
| Archirhodomyrtus beckleri 2022 | 4 | 15 | WB | 9 | 6 | 67 | |
| Archirhodomyrtus beckleri 2022 | 4 | 15 | WB | 10 | 8 | 80 | |
| Archirhodomyrtus beckleri 2022 | 4 | 15 | WB | 6 | 6 | 100 | |
| Archirhodomyrtus beckleri 2022 | 4 | 15 | WB | 8 | 7 | 88 | |
| Archirhodomyrtus beckleri 2022 | 4 | 15 | WB | 9 | 8 | 89 | 85 |
| Archirhodomyrtus beckleri 2022 | -5 | 15 | WB | 10 | 8 | 80 | |
| Archirhodomyrtus beckleri 2022 | -5 | 15 | WB | 9 | 5 | 56 | |
| Archirhodomyrtus beckleri 2022 | -5 | 15 | WB | 10 | 5 | 50 | |
| Archirhodomyrtus beckleri 2022 | -5 | 15 | WB | 10 | 8 | 80 | |
| Archirhodomyrtus beckleri 2022 | -5 | 15 | WB | 9 | 6 | 67 | 66 |
| Archirhodomyrtus beckleri 2022 | -20 | 15 | WB | 10 | 4 | 40 | |
| Archirhodomyrtus beckleri 2022 | -20 | 15 | WB | 10 | 3 | 30 | |
| Archirhodomyrtus beckleri 2022 | -20 | 15 | WB | 8 | 2 | 25 | |
| Archirhodomyrtus beckleri 2022 | -20 | 15 | WB | 11 | 2 | 18 | |
| Archirhodomyrtus beckleri 2022 | -20 | 15 | WB | 11 | 2 | 18 | 26 |
| Archirhodomyrtus beckleri 2022 | -192 | 15 | WB | 9 | 6 | 67 | |
| Archirhodomyrtus beckleri 2022 | -192 | 15 | WB | 9 | 2 | 22 | |
| Archirhodomyrtus beckleri 2022 | -192 | 15 | WB | 10 | 1 | 10 | |
| Archirhodomyrtus beckleri 2022 | -192 | 15 | WB | 10 | 0 | 0 | |
| Archirhodomyrtus beckleri 2022 | -192 | 15 | WB | 10 | 1 | 10 | 22 |
| Archirhodomyrtus beckleri 2022 | 4 | 50 | WB | 8 | 8 | 100 | |
| Archirhodomyrtus beckleri 2022 | 4 | 50 | WB | 9 | 5 | 56 | |
| Archirhodomyrtus beckleri 2022 | 4 | 50 | WB | 9 | 2 | 22 | |
| Archirhodomyrtus beckleri 2022 | 4 | 50 | WB | 8 | 7 | 88 | |
| Archirhodomyrtus beckleri 2022 | 4 | 50 | WB | 10 | 6 | 60 | 65 |
| Archirhodomyrtus beckleri 2022 | -5 | 50 | WB | 8 | 8 | 100 | |
| Archirhodomyrtus beckleri 2022 | -5 | 50 | WB | 9 | 8 | 89 | |
| Archirhodomyrtus beckleri 2022 | -5 | 50 | WB | 8 | 8 | 100 | |
| Archirhodomyrtus beckleri 2022 | -5 | 50 | WB | 11 | 11 | 100 | |
| Archirhodomyrtus beckleri 2022 | -5 | 50 | WB | 11 | 8 | 73 | 92 |
| Archirhodomyrtus beckleri 2022 | -20 | 50 | WB | 9 | 2 | 22 | |
| Archirhodomyrtus beckleri 2022 | -20 | 50 | WB | 10 | 4 | 40 | |
| Archirhodomyrtus beckleri 2022 | -20 | 50 | WB | 10 | 0 | 0 | |
| Archirhodomyrtus beckleri 2022 | -20 | 50 | WB | 10 | 3 | 30 | |
| Archirhodomyrtus beckleri 2022 | -20 | 50 | WB | 10 | 2 | 20 | 22 |
| Archirhodomyrtus beckleri 2022 | -192 | 50 | WB | 9 | 3 | 33 | |
| Archirhodomyrtus beckleri 2022 | -192 | 50 | WB | 9 | 8 | 89 | |

| Species | Storage temp. (°C) | RH (%) | Thawing temp. | No. sown | No. germ. | Germ (%) | Avg. Germ (%) |
|---|---|---|---|---|---|---|---|
| Archirhodomyrtus beckleri 2022 | -192 | 50 | WB | 10 | 3 | 30 | |
| Archirhodomyrtus beckleri 2022 | -192 | 50 | WB | 10 | 6 | 60 | |
| Archirhodomyrtus beckleri 2022 | -192 | 50 | WB | 6 | 3 | 50 | 52 |
| | | | | | | | |
| E. australe subsp. australe | 15 | 17 | RT | 11 | 9 | 82 | |
| E. australe subsp. australe | 15 | 17 | RT | 11 | 7 | 64 | |
| E. australe subsp. australe | 15 | 17 | RT | 11 | 11 | 100 | 82 |
| E. australe subsp. australe | -2 | 17 | RT | 11 | 11 | 100 | |
| E. australe subsp. australe | -2 | 17 | RT | 11 | 11 | 100 | |
| E. australe subsp. australe | -2 | 17 | RT | 11 | 11 | 100 | 100 |
| E. australe subsp. australe | -20 | 17 | RT | 11 | 10 | 91 | |
| E. australe subsp. australe | -20 | 17 | RT | 11 | 10 | 91 | |
| E. australe subsp. australe | -20 | 17 | RT | 11 | 11 | 100 | 94 |
| E. australe subsp. australe | 15 | 31 | RT | 11 | 11 | 100 | |
| E. australe subsp. australe | 15 | 31 | RT | 11 | 9 | 82 | |
| E. australe subsp. australe | 15 | 31 | RT | 11 | 11 | 100 | 94 |
| E. australe subsp. australe | -2 | 31 | RT | 11 | 7 | 64 | |
| E. australe subsp. australe | -2 | 31 | RT | 11 | 8 | 73 | |
| E. australe subsp. australe | -2 | 31 | RT | 11 | 9 | 82 | 79 |
| E. australe subsp. australe | -20 | 31 | RT | 11 | 11 | 100 | |
| E. australe subsp. australe | -20 | 31 | RT | 11 | 10 | 91 | |
| E. australe subsp. australe | -20 | 31 | RT | 11 | 11 | 100 | 91 |
| | | | | | | | |
| Emmenosperma alphitonioides | 15 | 18 | RT | 10 | 3 | 30 | |
| Emmenosperma alphitonioides | 15 | 18 | RT | 10 | 9 | 90 | |
| Emmenosperma alphitonioides | 15 | 18 | RT | 10 | 10 | 100 | |
| Emmenosperma alphitonioides | 15 | 18 | RT | 10 | 10 | 100 | |
| Emmenosperma alphitonioides | 15 | 18 | RT | 10 | 5 | 50 | 74 |
| Emmenosperma alphitonioides | 4 | 18 | RT | 10 | 9 | 90 | |
| Emmenosperma alphitonioides | 4 | 18 | RT | 10 | 6 | 60 | |
| Emmenosperma alphitonioides | 4 | 18 | RT | 10 | 3 | 30 | |
| Emmenosperma alphitonioides | 4 | 18 | RT | 10 | 6 | 60 | |
| Emmenosperma alphitonioides | 4 | 18 | RT | 10 | 6 | 60 | 60 |
| Emmenosperma alphitonioides | -2 | 18 | RT | 10 | 7 | 70 | |
| Emmenosperma alphitonioides | -2 | 18 | RT | 10 | 8 | 80 | |
| Emmenosperma alphitonioides | -2 | 18 | RT | 10 | 9 | 90 | |
| Emmenosperma alphitonioides | -2 | 18 | RT | 10 | 7 | 70 | |
| Emmenosperma alphitonioides | -2 | 18 | RT | 10 | 10 | 100 | 82 |
| Emmenosperma alphitonioides | -20 | 18 | RT | 10 | 9 | 90 | |
| Emmenosperma alphitonioides | -20 | 18 | RT | 10 | 9 | 90 | |
| Emmenosperma alphitonioides | -20 | 18 | RT | 10 | 8 | 80 | |
| Emmenosperma alphitonioides | -20 | 18 | RT | 10 | 5 | 50 | |
| Emmenosperma alphitonioides | -20 | 18 | RT | 10 | 5 | 50 | 72 |
| Emmenosperma alphitonioides | 15 | 30 | RT | 9 | 5 | 56 | |
| Emmenosperma alphitonioides | 15 | 30 | RT | 10 | 7 | 70 | |

| Species | Storage temp. (°C) | RH (%) | Thawing temp. | No. sown | No. germ. | Germ (%) | Avg. Germ (%) |
|---|---|---|---|---|---|---|---|
| Emmenosperma alphitonioides | 15 | 30 | RT | 10 | 6 | 60 | |
| Emmenosperma alphitonioides | 15 | 30 | RT | 10 | 8 | 80 | |
| Emmenosperma alphitonioides | 15 | 30 | RT | 10 | 8 | 80 | 69 |
| Emmenosperma alphitonioides | 4 | 30 | RT | 10 | 6 | 60 | |
| Emmenosperma alphitonioides | 4 | 30 | RT | 10 | 8 | 80 | |
| Emmenosperma alphitonioides | 4 | 30 | RT | 10 | 8 | 80 | |
| Emmenosperma alphitonioides | 4 | 30 | RT | 10 | 8 | 80 | |
| Emmenosperma alphitonioides | 4 | 30 | RT | 10 | 8 | 80 | 76 |
| Emmenosperma alphitonioides | -2 | 30 | RT | 10 | 1 | 10 | |
| Emmenosperma alphitonioides | -2 | 30 | RT | 10 | 8 | 80 | |
| Emmenosperma alphitonioides | -2 | 30 | RT | 10 | 6 | 60 | |
| Emmenosperma alphitonioides | -2 | 30 | RT | 10 | 6 | 60 | |
| Emmenosperma alphitonioides | -2 | 30 | RT | 10 | 2 | 20 | 46 |
| Emmenosperma alphitonioides | -20 | 30 | RT | 10 | 6 | 60 | |
| Emmenosperma alphitonioides | -20 | 30 | RT | 10 | 6 | 60 | |
| Emmenosperma alphitonioides | -20 | 30 | RT | 10 | 9 | 90 | |
| Emmenosperma alphitonioides | -20 | 30 | RT | 10 | 9 | 90 | |
| Emmenosperma alphitonioides | -20 | 30 | RT | 10 | 5 | 50 | 70 |
| | | | | | | | |
| Melastoma affine | -20 | 15 | RT | 10 | 1 | 10 | |
| Melastoma affine | -20 | 15 | RT | 10 | 1 | 10 | |
| Melastoma affine | -20 | 15 | RT | 10 | 0 | 0 | |
| Melastoma affine | -20 | 15 | RT | 10 | 2 | 20 | |
| Melastoma affine | -20 | 15 | RT | 10 | 0 | 0 | 8 |
| Melastoma affine | -192 | 15 | RT | 10 | 5 | 50 | |
| Melastoma affine | -192 | 15 | RT | 10 | 4 | 40 | |
| Melastoma affine | -192 | 15 | RT | 10 | 6 | 60 | |
| Melastoma affine | -192 | 15 | RT | 10 | 6 | 60 | |
| Melastoma affine | -192 | 15 | RT | 10 | 9 | 90 | 60 |
| | | | | | | | |
| Pittosporum multiflorum | 15 | 17 | RT | 10 | 9 | 90 | |
| Pittosporum multiflorum | 15 | 17 | RT | 10 | 10 | 100 | |
| Pittosporum multiflorum | 15 | 17 | RT | 10 | 9 | 90 | |
| Pittosporum multiflorum | 15 | 17 | RT | 10 | 10 | 100 | 95 |
| Pittosporum multiflorum | -5 | 17 | RT | 10 | 9 | 90 | |
| Pittosporum multiflorum | -5 | 17 | RT | 10 | 10 | 100 | |
| Pittosporum multiflorum | -5 | 17 | RT | 10 | 10 | 100 | |
| Pittosporum multiflorum | -5 | 17 | RT | 9 | 9 | 100 | 98 |
| Pittosporum multiflorum | -20 | 17 | RT | 10 | 7 | 70 | |
| Pittosporum multiflorum | -20 | 17 | RT | 10 | 7 | 70 | |
| Pittosporum multiflorum | -20 | 17 | RT | 10 | 6 | 60 | |
| Pittosporum multiflorum | -20 | 17 | RT | 10 | 2 | 20 | 55 |
| Pittosporum multiflorum | 15 | 28 | RT | 10 | 10 | 100 | |
| Pittosporum multiflorum | 15 | 28 | RT | 10 | 9 | 90 | |
| Pittosporum multiflorum | 15 | 28 | RT | 10 | 9 | 90 | |

| Species | Storage temp. (°C) | RH (%) | Thawing temp. | No. sown | No. germ. | Germ (%) | Avg. Germ (%) |
|---|---|---|---|---|---|---|---|
| Pittosporum multiflorum | 15 | 28 | RT | 10 | 9 | 90 | 93 |
| Pittosporum multiflorum | -5 | 28 | RT | 10 | 9 | 90 | |
| Pittosporum multiflorum | -5 | 28 | RT | 10 | 7 | 70 | |
| Pittosporum multiflorum | -5 | 28 | RT | 10 | 9 | 90 | |
| Pittosporum multiflorum | -5 | 28 | RT | 10 | 10 | 100 | 88 |
| Pittosporum multiflorum | -20 | 28 | RT | 9 | 3 | 33 | |
| Pittosporum multiflorum | -20 | 28 | RT | 10 | 4 | 40 | |
| Pittosporum multiflorum | -20 | 28 | RT | 10 | 1 | 10 | |
| Pittosporum multiflorum | -20 | 28 | RT | 10 | 1 | 10 | 23 |
| | | | | | | | |
| Rhodamnia maideniana | 4 | 15 | RT | 10 | 10 | 100 | |
| Rhodamnia maideniana | 4 | 15 | RT | 10 | 9 | 90 | |
| Rhodamnia maideniana | 4 | 15 | RT | 10 | 10 | 100 | |
| Rhodamnia maideniana | 4 | 15 | RT | 10 | 10 | 100 | |
| Rhodamnia maideniana | 4 | 15 | RT | 10 | 9 | 90 | 96 |
| Rhodamnia maideniana | -5 | 15 | RT | 10 | 9 | 90 | |
| Rhodamnia maideniana | -5 | 15 | RT | 10 | 9 | 90 | |
| Rhodamnia maideniana | -5 | 15 | RT | 10 | 10 | 100 | |
| Rhodamnia maideniana | -5 | 15 | RT | 10 | 10 | 100 | |
| Rhodamnia maideniana | -5 | 15 | RT | 10 | 8 | 80 | 92 |
| Rhodamnia maideniana | -20 | 15 | RT | 10 | 1 | 10 | |
| Rhodamnia maideniana | -20 | 15 | RT | 10 | 0 | 0 | |
| Rhodamnia maideniana | -20 | 15 | RT | 10 | 2 | 20 | |
| Rhodamnia maideniana | -20 | 15 | RT | 10 | 3 | 30 | |
| Rhodamnia maideniana | -20 | 15 | RT | 10 | 2 | 20 | 16 |
| Rhodamnia maideniana | -192 | 15 | RT | 10 | 4 | 40 | |
| Rhodamnia maideniana | -192 | 15 | RT | 10 | 6 | 60 | |
| Rhodamnia maideniana | -192 | 15 | RT | 10 | 6 | 60 | |
| Rhodamnia maideniana | -192 | 15 | RT | 10 | 6 | 60 | |
| Rhodamnia maideniana | -192 | 15 | RT | 10 | 6 | 60 | 56 |
